# mRNA PROTACs: engineering PROTACs for high‐efficiency targeted protein degradation

**DOI:** 10.1002/mco2.478

**Published:** 2024-02-19

**Authors:** Xiaoqi Xue, Chen Zhang, Xiaolin Li, Junqiao Wang, Haowei Zhang, Ying Feng, Naihan Xu, Hongyan Li, Chunyan Tan, Yuyang Jiang, Ying Tan

**Affiliations:** ^1^ State Key Laboratory of Chemical Oncogenomics Institute of Biopharmaceutical and Health Engineering Shenzhen International Graduate School, Tsinghua University Shenzhen China; ^2^ School of Food and Drug Shenzhen Polytechnic University Shenzhen China; ^3^ Shenzhen NeoCura Biotechnology Co., Ltd. Shenzhen China

**Keywords:** *in vitro* transcribed mRNA (IVT‐mRNA), mRNA, peptide proteolysis‐argeting chimeras, proteolysis‐argeting chimeras (PROTAC), Targeted protein degradation (TPD)

## Abstract

Proteolysis‐targeting chimeras (PROTACs) are essential bifunctional molecules that target proteins of interest (POIs) for degradation by cellular ubiquitination machinery. Despite significant progress made in understanding PROTACs' functions, their therapeutic potential remains largely untapped. As a result of the success of highly flexible, scalable, and low‐cost mRNA therapies, as well as the advantages of the first generation of peptide PROTACs (p‐PROTACs), we present for the first time an engineering mRNA PROTACs (m‐PROTACs) strategy. This design combines von Hippel–Lindau (VHL) recruiting peptide encoding mRNA and POI‐binding peptide encoding mRNA to form m‐PROTAC and promote cellular POI degradation. We then performed proof‐of‐concept experiments using two m‐PROTACs targeting two cancer‐related proteins, estrogen receptor alpha and B‐cell lymphoma‐extra large protein. Our results demonstrated that m‐PROTACs could successfully degrade the POIs in different cell lines and more effectively inhibit cell proliferation than the traditional p‐PROTACs. Moreover, the in vivo experiment demonstrated that m‐PROTAC led to significant tumor regression in the 4T1 mouse xenograft model. This finding highlights the enormous potential of m‐PROTAC as a promising approach for targeted protein degradation therapy.

## Graphical abstract

We developed mRNA proteolysis‐targeting chimeras (PROTACs), a novel type of PROTACs, which incorporate mRNAs encoding E3 ligase recruiting peptide ligand and protein of interest (POI)‐binding peptide ligand to promote cellular POI degradation. For the first time, we demonstrated that the encoding peptide PROTACs could degrade POIs and inhibit cell proliferation more effectively than the traditional peptide PROTACs, indicating their tremendous potential for targeted protein degradation therapy.

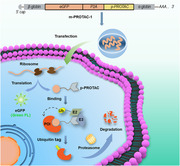

## 1 INTRODUCTION

Targeted protein degradation (TPD) is an emerging strategy that is currently used to shut down the functions of specific proteins in living cells. TPD drugs developed to date include proteolysis‐targeting chimeras (PROTACs), which are bifunctional molecules that usually consist of three basic elements: a ligand that specifically recruits a cellular target protein, a second ligand that recruits a cellular E3 ligase, and a linker connecting the two ligands together.^1^ To perform their therapeutic function, PROTACs promote cellular E3 ligase activity that targets specific cellular proteins for proteasomal hydrolysis by the cellular ubiquitin–proteasome system (UPS).^2^ As compared with traditional small‐molecule inhibitors (SMIs) that block protein functions by occupying protein active sites, PROTACs bring target proteins into close proximity with cellular E3 ligase molecules that ultimately remove targeted proteins from cells via a process that mainly relies on ligand‐target binding affinities, not on blocking of protein function. Therefore, PROTACs avoid SMI‐associated drug resistance by acting on novel undruggable targets.^3^ In 2001, the concept of PROTACs for use in TPD was first proposed by Crews and coworkers,^4^ after which research interest in TPD has increased dramatically. Early PROTACs mainly consisted of combinations of peptides and/or small molecules that have since been replaced by various types of molecules, resulting in the development of diverse types of PROTACs.^5^, ^6^, ^7^, ^8^, ^9^, ^10^

The newest wave of PROTACs development has focused on PROTACs designs that have improved PROTACs cellular permeability, biocompatibility, and safety.^6^, ^11^, ^12^ BioPROTACs are composed of biomolecules, such as peptides or nucleic acids, instead of small molecules. These biomolecules offer high specificity and biocompatibility, but often face challenges in terms of cell permeation. However, these challenges can be addressed by utilizing mRNA or dye used to localize the nucleus of cells (DNA) delivery systems.^1^ One of their greatest advantages is their capability to target undruggable proteins,^13^ so newer PROTAC designs will allow new molecules and targets to be developed.

Eleven years before PROTACs were discovered, Wolff et al. put forth the idea of “in vitro transcribed mRNA” (IVT‐mRNA) as a potential therapeutic treatment that has gradually attracted the interest of the research community.^14^ Since that time, researchers have overcome challenges related to mRNA instability (half‐life), inefficient translation and high immunogenicity by modifying mRNAs in various ways.^15^, ^16^ Such modifications have involved alterations of mRNA 5′ cap, 5′ and 3′ untranslated regions (UTRs), 3′ poly(A) tail and coding sequences, as well as replacements of rare codons with frequent codons and insertions of aminoacyl tRNA repeats.^17^, ^18^ In eukaryotic cells, natural mRNA contains a 7‐methyl guanosine (m7G) cap that enhances the efficiency of translation by binding to the translation initiation factor 4E (EIF4E). Additionally, it is worth noting that the binding of mRNA decapping enzymes to the 5′ cap can have an impact on mRNA degradation and subsequently reduce translation efficiency.^19^ This highlights the significance of maintaining a functional 5′ cap structure for ensuring stable mRNA translation.^20^ In the process of gene expression, the UTR of mRNA plays a crucial role in regulating various aspects of mRNA function, including its export from the nucleus, translation efficiency and stability, and subcellular localization. For instance, incorporating the 3′ UTR of α‐globin can enhance mRNA stability, while the inclusion of both 5′ and 3′ UTRs of β‐globin can improve mRNA translation efficiency.^21^ Additionally, the presence of a poly(A) tail at the end of the mRNA sequence has been shown to increase efficiency and prevent mRNA degradation.^22^, ^23^ These discoveries have enhanced IVT‐mRNA functionality by leaps and bounds in recent years and led to the development of diverse mRNA‐based therapeutics and vaccines.^24^ Thus, researchers have only begun to tap the potential of mRNA therapy as a highly flexible, scalable, and low‐cost drug development platform.

In this study, we first proposed the engineering strategy of mRNA PROTACs (referred to hereafter as m‐PROTACs), which was a novel type of PROTACs combining peptide PROTACs (referred to hereafter as p‐PROTACs) with IVT‐mRNA molecules. m‐PROTACs are mRNA molecules that are obtained through IVT, delivered into cells, and subsequently translated to their corresponding p‐PROTAC form. The p‐PROTAC is composed of an E3 recruiting peptide ligand and a protein of interest (POI)‐binding peptide ligand, which work together to promote the degradation of the target POI via the cellular UPS system. As proof of strategy, we applied this engineering strategy to achieve cellular removal of typical target proteins, including estrogen receptor alpha (ERα) and B‐cell lymphoma‐extra large protein (BCL‐x_L_), then evaluated our m‐PROTACs for antitumor activity. Our results demonstrated effective m‐PROTAC‐induced removal of targeted proteins from different cells, as well as enhanced cell proliferation inhibition in vitro and in vivo. m‐PROTACs owns the character of high safety and biocompatibility which would be part of BioPROTACs. Importantly, as compared to the traditional p‐PROTACs, the new m‐PROTACs enabled the administration of less‐immunogenic lower doses of m‐PROTACs to achieve the same TPD effect while utilizing the selectivity and biocompatibility of p‐PROTACs. Due to these advantages, m‐PROTACs also hold promise as novel mRNA therapeutics for use in treating human diseases.

## 2 RESULTS

### 2.1 Design of m‐PROTAC‐1

**FIGURE 1:**
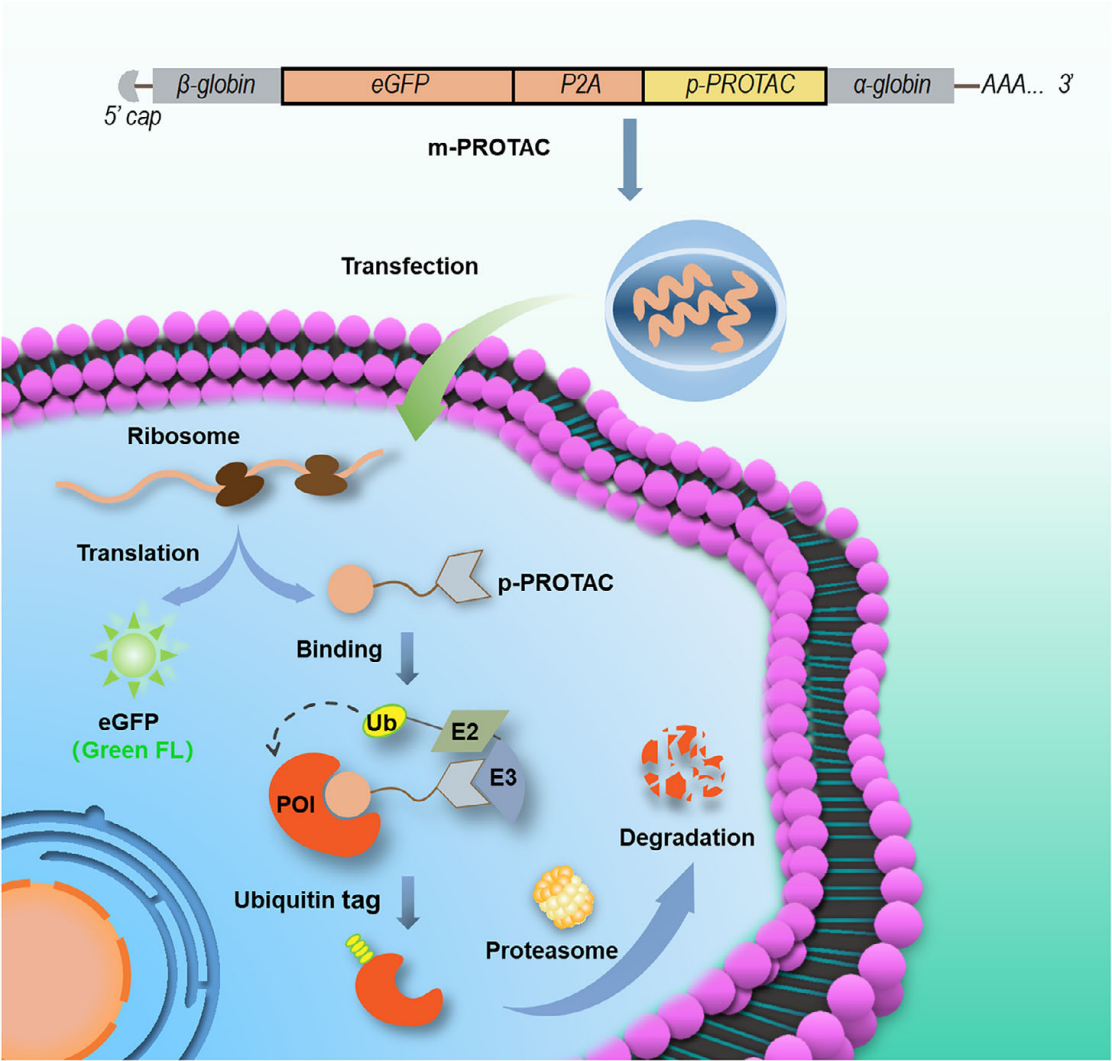
A schematic diagram of mRNA proteolysis‐targeting chimera (m‐PROTAC) strategy. m‐PROTAC was synthesized by in vitro transcribed mRNA (IVT‐mRNA) and transfected into cells to encode enhanced green fluorescent protein (eGFP) and peptide PROTAC (p‐PROTAC). Encoding p‐PROTAC can mediate the ubiquitination and degradation of protein of interest (POI).

ERα, a transcription factor that modulates cell proliferation, is a nuclear ER. Notably, ERα is overexpressed by most types of breast cancer cells and thus is frequently targeted by breast cancer treatments.^10^ For example, Li's research group synthesized a p‐PROTAC containing an ERα‐targeting peptide that successfully removed ERα from cells both in vivo and in vitro.^25^ Similarly, here an encoding p‐PROTAC was designed based on the ERα peptide ligand (QLLRHLILH) mentioned in the above report and the peptide ligand (PIYPALA) recognized by the Von Hippel–Lindau (VHL) E3 ligase, which has been successfully used in a variety of peptide‐based PROTACs. Both of the target peptide ligands were joined together with the classical linker peptide GSGS between them to generate the complete peptide sequence (PIYPALAGSGSQLLRHLILH). Next, we designed an mRNA sequence based on this peptide to generate the m‐PROTAC targeting ERα (m‐PROTAC‐1). In order to verify that m‐PROTAC‐1 could be expressed by cells, we ligated an enhanced green fluorescent protein (eGFP)‐encoding sequence in the front of the m‐PROTAC‐1 sequence. Additionally, the mRNA sequence encoding P2A element (GSGATNFSLLKQAGDVEENPGP), a reported tool of cleavage between the genes upstream and downstream, was used to ligate eGFP and the p‐PROTAC sequences.^26^ During the translation of the m‐PROTAC‐1 sequence, ribosomal translation of the eGFP‐P2A element sequences occurs until the ribosome reaches the final P2A glycine and proline amino acids, at which point the ribosome is unable to form a peptide bond.^27^ The ribosome then continues to the adjacent downstream sequence after leaving a gap between the translated eGFP‐P2A and the p‐PROTAC, resulting in their synthesis as two separate parts. In order to increase the translation efficiency and stability of m‐PROTAC‐1, it was further modified by adding human β‐globin 5′ UTR and human α‐globin 3′ UTR sequences to its 5′ and 3′ ends, respectively, while adding 5′ m7G cap and 3′ poly‐A tail sequences to its mRNA sequence (Figure 1). Introducing the 3′ UTR of α‐globin can improve the stability of mRNA, while incorporation of 5′ and 3′ UTRs of β‐globin can improve translation efficiency.

Theoretically, m‐PROTAC‐1 is capable of harnessing the cellular translational machinery to direct cellular synthesis of functional protein‐targeting p‐PROTAC and a separate eGFP protein for use in verifying peptide expression. Once the encoding p‐PROTAC is synthesized by the cell, they can interact with endogenous ERα and VHL to trigger cellular UPS‐driven ERα degradation on site (Figure 1).

### 2.2 Effect of m‐PROTAC‐1 on ERα degradation

**FIGURE 2:**
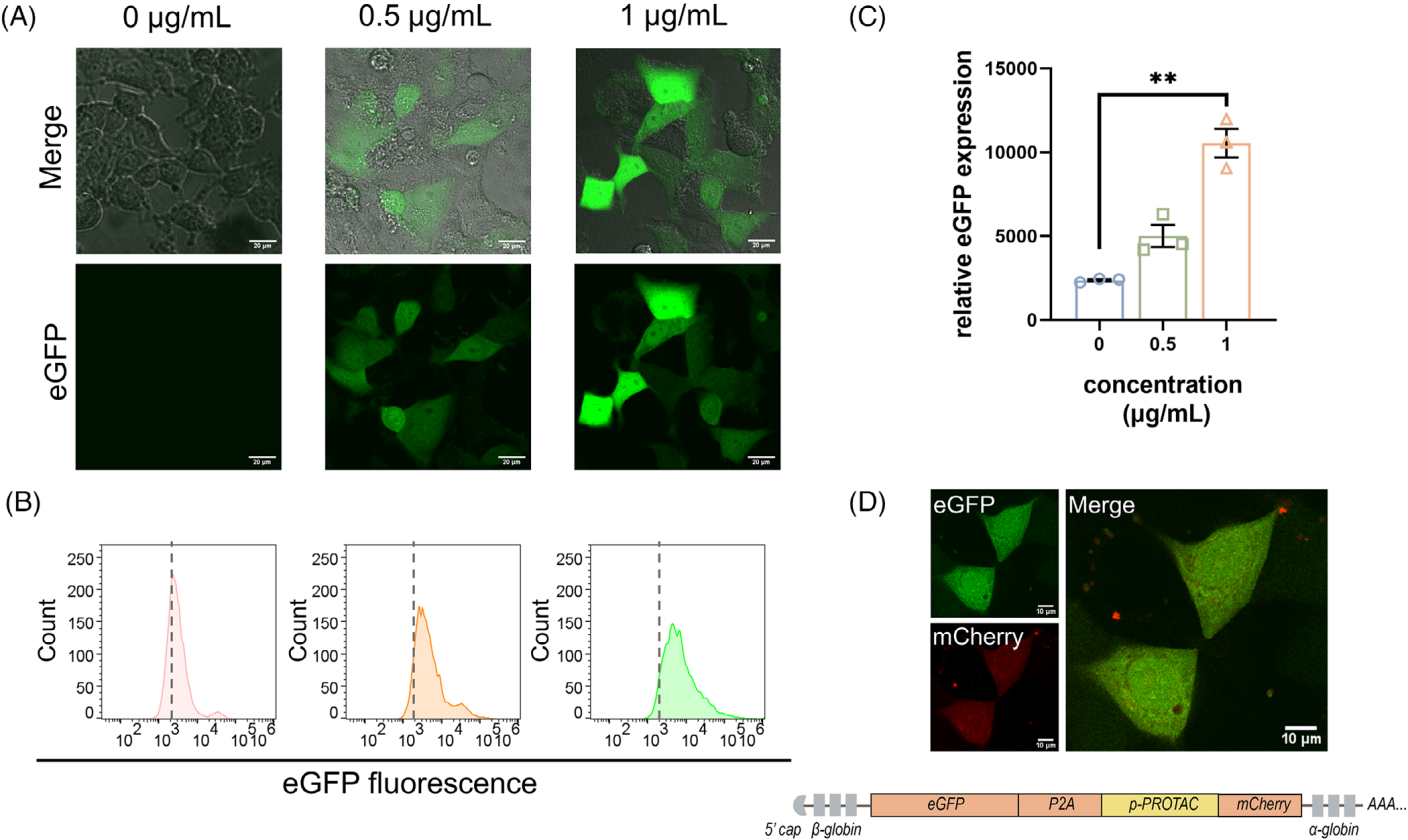
mRNA proteolysis‐targeting chimera (m‐PROTAC)‐1 showed stable translation efficiency in MCF‐7 cells. (A) Confocal microscopy images and (B) flow cytometry analysis (histogram) of MCF‐7 cells transfected with the indicated concentrations of m‐PROTAC‐1 for 24 h (scale bar, 20 μm). (C) Quantification of relative enhanced green fluorescent protein (eGFP) expression level of MCF‐7 cells from (B). (D) Confocal microscopy images of MCF‐7 cells treated with 1 μg/mL of mCherry added mRNA for 24 h (scale bar, 10 μm). Data are presented as mean ± SEM of triplicate independent experiments for (B). *n* = 3; ^*^ *p* < 0.05; ^**^ *p* < 0.01.

After synthesizing m‐PROTAC‐1 by IVT‐mRNA technology (Figure S1), we assessed the expression of the p‐PROTAC firstly in the ERα‐positive breast cancer cell line Michigan Cancer Foundation‐7 (MCF‐7) cells. After the cells were transfected with 0, 0.5, or 1 μg/mL m‐PROTAC‐1, they were cultured for 24 h then assessed for dose‐dependent eGFP expression using cell imaging and flow cytometry. After culture, obvious green fluorescence was observed that revealed dose‐dependent cellular expression of eGFP, indicating that p‐PROTAC were translated with high efficiency (Figure 2A–C). We also constructed another mRNA containing extra mCherry red fluorescent protein sequence following p‐PROTAC sequence for the use as a reporter protein. As shown in Figure 2D, overlapping strong signals related to eGFP‐induced green fluorescence and mCherry‐induced red fluorescence were observed in cells transfected with the mCherry added mRNA, thus demonstrating stable and efficient translation of m‐PROTAC‐1 by MCF‐7 cells directly.

Next, we investigated whether the lipo6000 transfection reagent used in our protocol affected the cellular ERα level. Figure S2 shows that ERα levels in MCF‐7 cells treated only with lipo6000 were similar to corresponding levels observed in untreated cells, while a significantly lower ERα level was observed in cells transfected with 1 μg/mL m‐PROTAC‐1. When MCF‐7 cells were treated with m‐PROTAC‐1 for indicated time, the enhancement of green fluorescent (eGFP) signal indicated an increased expression of p‐PROTAC within the cells, while the weakening of the red fluorescence (Cy5‐labeled ERα antibody) signal suggested that the ERα is being induced for degradation with the generation of p‐PROTAC (Figure S3).

**FIGURE 3:**
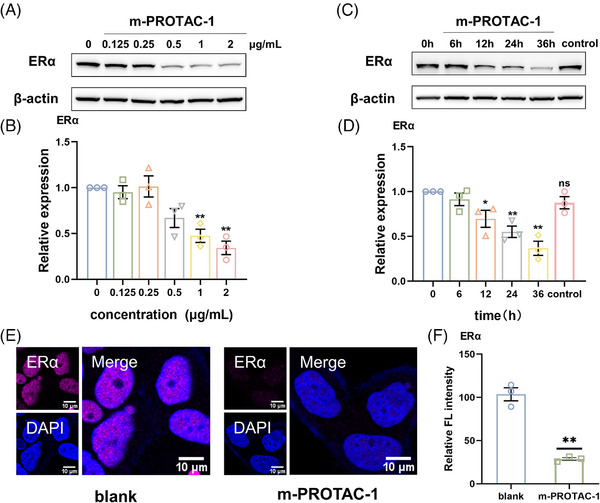
mRNA proteolysis‐targeting chimera (m‐PROTAC)‐1 induced the degradation of estrogen receptor alpha (ERα). (A) Western blot of ERα in MCF‐7 cells after transfected with the indicated concentrations of m‐PROTAC‐1 for 24 h. (B) Quantification of expression levels of ERα of MCF‐7 cells from (A). (C) Western blot of ERα in MCF‐7 cells after transfected with 1 μg/mL m‐PROTAC‐1 for different lengths of time. (D) Quantification of expression levels of ERα of MCF‐7 cells from (C). (E) Confocal microscopy images of MCF‐7 cells treated with 1 μg/mL m‐PROTAC‐1 for 24 h and stained with labeled ERα antibody (scale bar, 10 μm). (F) Quantification of imaging data of MCF‐7 cells from (E). Data are presented as mean ± SEM of triplicate independent experiments for (B), (D), and (F). *n* = 3; ns, *p* > 0.05; ^*^ *p* < 0.05; ^**^ *p* < 0.01.

To evaluate the kinetics of the TPD process, we transfected MCF‐7 cells with different m‐PROTAC‐1 concentrations (0, 0.125, 0.25, 0.5, 1, and 2 μg/mL) or incubated transfected cells for various amounts of time then assessed intracellular ERα levels via western blotting. The results revealed dose‐dependent reductions in intracellular ERα levels, with a TPD efficiency observed of 50% for cells transfected with m‐PROTAC‐1 at a concentration of 1 μg/mL after 24 h culture (Figure 3A,B). The working concentration was equal to 3 nM, exhibiting obvious dose advantage compared with other reported PROTACs targeting ERα, most of which were usually μM level.^10^, ^25^, ^28^ Moreover, m‐PROTAC‐1 induced intracellular ERα degradation in a time‐dependent manner, as revealed by results obtained after transfected cells were cultured for various amounts of time (0, 6, 12, 24, or 36 h), with the ERα level dropping off sharply after 24 h of culture. Importantly, the control group transfected with 1 μg/mL of mRNA that lacked encoding p‐PROTAC sequence exhibited no ERα degradation (Figure 3C,D). To further verify that m‐PROTAC‐1 induced cellular ERα degradation, immunofluorescence assays were conducted. Results of these experiments revealed that red fluorescence that was emitted by cy5‐labeled ERα in nuclei of untreated control cells was significantly reduced in that of m‐PROTAC‐1‐treated cells, as consistent with results of quantitative fluorescence analysis (Figure 3E,F). The above results demonstrated that m‐PROTAC‐1, a novel type of PROTAC, could efficiently induce degradation of ERα in MCF‐7 cells, as confirmed by results of similar experiments conducted using T47D cells, another breast cancer‐derived cell line (Figure S3).

### 2.3 Evaluation of the mechanism of m‐PROTAC‐1

**FIGURE 4:**
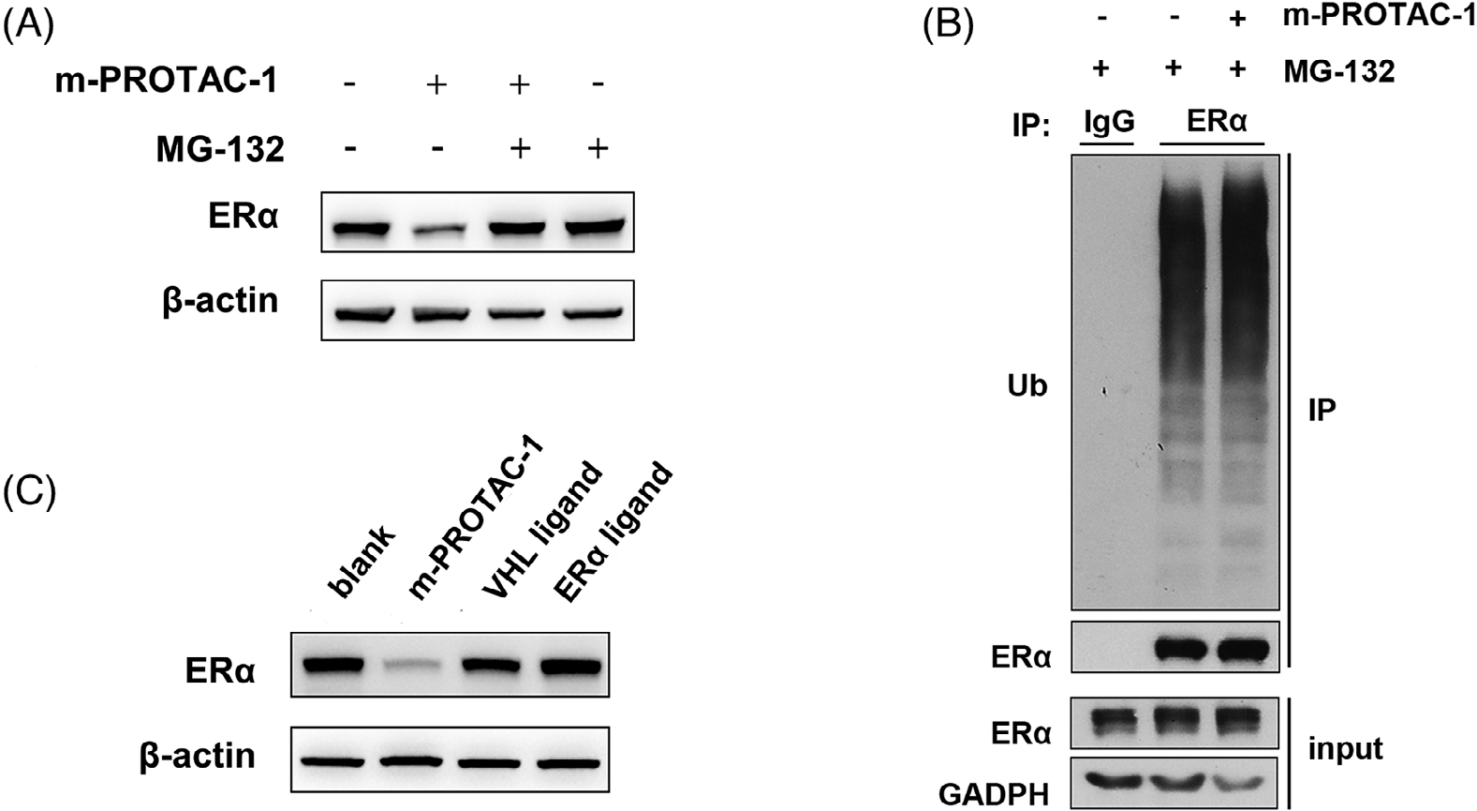
mRNA proteolysis‐targeting chimera (m‐PROTAC)‐1 induces estrogen receptor alpha (ERα) degradation in a proteasome‐dependent manner. (A) Western blot of ERα in MCF‐7 cells after transfected with 1 μg/mL m‐PROTAC‐1 with or without 10 μM MG‐132 for 24 h. (B) Co‐immunoprecipitation analysis of the ubiquitination of endogenous ERα proteins in MCF‐7 cells 24 h after transfection with 1 μg/mL m‐PROTAC‐1 and 10 μM MG‐132. (C) Western blot of ERα in MCF‐7 cells after treatment with 1 μg/mL m‐PROTAC‐1 or 20 μM peptide ligands for 24 h. Data are presented as mean ± SEM of triplicate independent experiments. *n* = 3; ns, *p* > 0.05; ^*^ *p* < 0.05.

Also of note, m‐PROTAC‐1 exerted its TPD effect by triggering cellular UPS activity, different from the mechanism used by other ERα antagonists, since treatment of MCF‐7 cells with the proteasome inhibitor MG‐132 (10 μM) or NEDD8‐activating enzyme inhibitor MLN4924 (0.5 μM) abolished the m‐PROTAC‐1 induced decrease in ERα level (Figures 4A, S4, and S5). In order to explore the ubiquitination of ERα, a co‐immunoprecipitation assay was conducted on MCF‐7 cells treated with m‐PROTAC‐1 and MG‐132, using an anti‐ERα antibody. The presence of 1 μg/mL m‐PROTAC‐1 and 10 μM MG‐132 resulted in an observable increase in ubiquitinated ERα, indicating that the degradation of ERα was indeed dependent on UPS (Figure 4B).

Moreover, neither ERα‐binding peptide ligand alone nor the VHL‐binding ligand alone could induce TPD of ERα (Figure 4C), while 20 μM is a much higher concentration than the 1 μg/mL of m‐PROTAC‐1 which was equal to 3 nM, demonstrating that the ternary complex was formed and played an essential role in protein degradation. Taken together, the above‐mentioned results indicated that m‐PROTAC‐1 behaved as expected by exerting a fantastic ERα‐specific degradative effect via UPS with the DC50 (the concentration that induces 50% protein degradation) achieving a surpassing nM level.

### 2.4 Effect of m‐PROTAC‐1 on MCF‐7 cells

**FIGURE 5:**
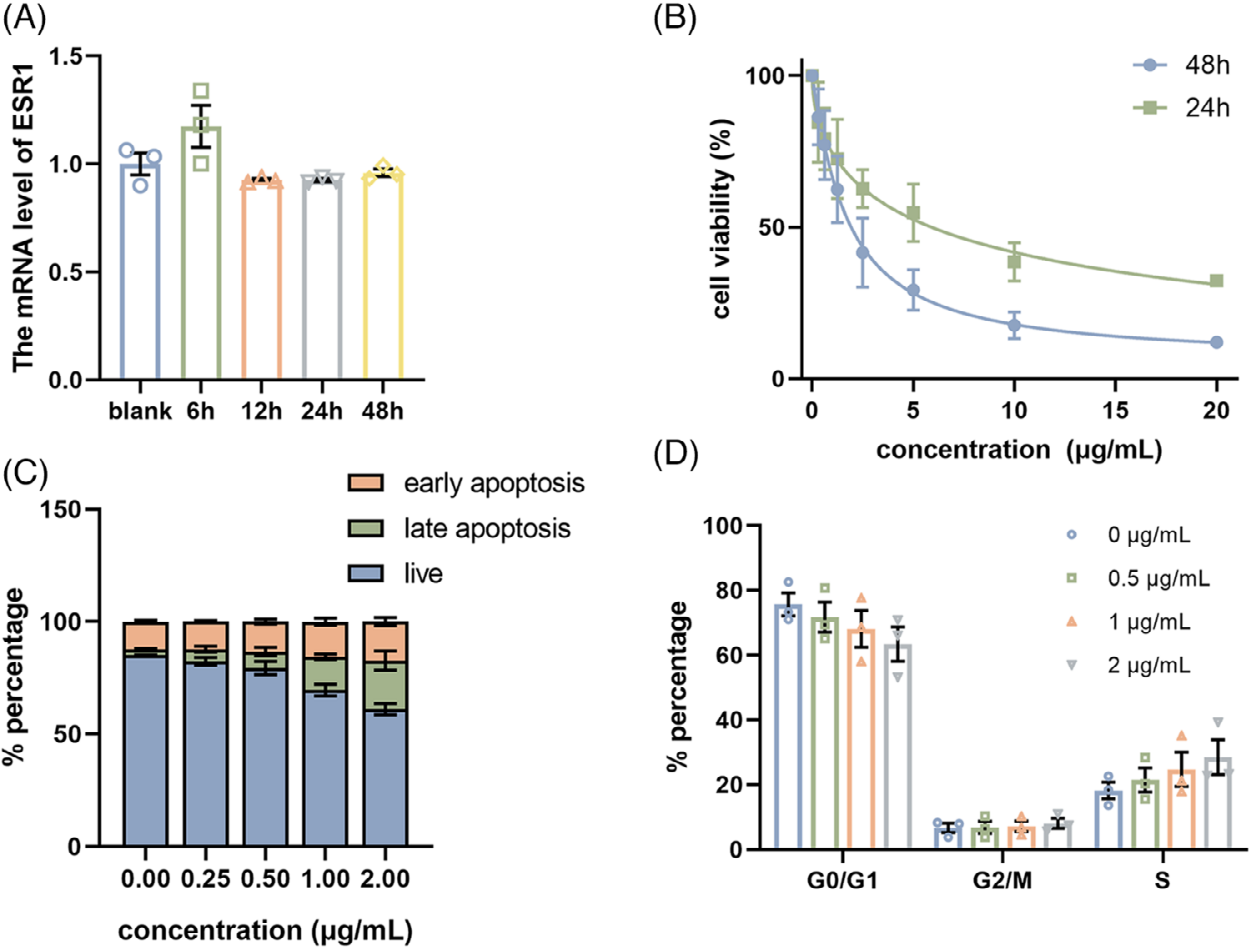
mRNA proteolysis‐targeting chimera (m‐PROTAC)‐1 treatment resulted in cell proliferation in MCF‐7 cells. (A) real‐time polymerase chain reaction (RT‐PCR) analysis of estrogen receptor alpha (ERα) genes in MCF‐7 cells after transfection with 1 μg/mL m‐PROTAC‐1 for 6, 12, 24, and 48 h (*n* = 3). (B) Antiproliferative effects of m‐PROTAC‐1 in the MCF‐7 cells, measured by Cell Counting Kit‐8 (CCK‐8) assay after transfected with different concentrations of m‐PROTAC‐1 for 24 or 48 h. (C) Percentage of apoptosis MCF‐7 cells, analyzed by Annexin V/propidium iodide (PI) staining after transfected with different concentrations of m‐PROTAC‐1 for 24 h. (D) Percentage of MCF‐7 cells in different phases of the cell cycle, analyzed by PI staining after transfected with different concentrations of m‐PROTAC‐1 for 24 h. The data are expressed as the mean ± SEM of triple independent experiments. *n* = 3; ^*^ *p* < 0.05; ^**^ *p* < 0.01; ^***^ *p* < 0.005.

We next analyzed the effect of m‐PROTAC‐1 on cell proliferation. Initially, the mRNA level of the ERα gene in MCF‐7 cells was assessed using quantitative real‐time polymerase chain reaction (qRT‐PCR) at various time points following treatment with m‐PROTAC‐1. The results indicated that m‐PROTAC‐1 effectively downregulated the expression of ERα while maintaining the mRNA level unchanged (Figure 5A). Then, Cell Counting Kit‐8 (CCK‐8) assays were performed by transfecting MCF‐7 cells with various concentrations of m‐PROTAC‐1 for 24 and 48 h. The results revealed that m‐PROTAC‐1 inhibited MCF‐7 cells growth (Figure 5B), as evidenced by IC50 values obtained for cells transfected with m‐PROTAC‐1 was at concentrations of approximately 8.2 μg/mL for 24 h and 1.7 μg/mL for 48 h (equal to 24.5 and 5.1 nM, respectively). Notably, the nM‐level IC50 value obtained for m‐PROTAC‐1 was much lower than the μM‐level IC50 values that were reported for ERα‐targeting p‐PROTACs.^25^, ^29^ To determine the mechanism underlying the observed decreased proliferation, apoptosis assays were performed by staining m‐PROTAC‐1‐transfected cells with Annexin V and propidium iodide (PI) after 24 h culture followed by flow cytometric analysis. A higher number of cells showed apoptotic features after high‐dose treatment than after low dose, indicating m‐PROTAC‐1 effectively induced apoptosis of MCF‐7 cells (Figures 5C and S7). Meanwhile, PI staining of m‐PROTAC‐1‐transfected cells after 24 h culture revealed that greater numbers of treated cells were arrested in S‐phase as compared to controls, thus suggesting that m‐PROTAC‐1 inhibited cell proliferation by blocking S‐phase cell‐cycle progression (Figures 5D and S8).

### 2.5 Design and evaluation of m‐PROTAC‐2

Due to the fact that BCL‐x_L_, a member of the BCL‐2 protein family, is viewed as another important cancer therapy target.^30^, ^31^, ^32^, ^33^ The second m‐PROTAC, m‐PROTAC‐2, was designed to encode a BCL‐x_L_‐targeting functional p‐PROTAC (PIYPALAGGGGGGGQVGRQLAIIGDAINR) connecting VHL ligase and BCL‐x_L_ simultaneously, which was reported by our previous work.^34^ Following the engineering design principles of m‐PROTAC‐1, m‐PROTAC‐2 only involved the substitution of the sequence encoding the corresponding p‐PROTAC. m‐PROTAC‐2 showed stable translation efficiency in MDA‐MB‐231 cells in Figure S9. In order to verify the ability of m‐PROTAC‐2 to induce cellular degradation of BCL‐x_L,_ different cancer cell lines were transfected with 1 μg/mL m‐PROTAC‐2 for 24 h. The results (Figure S10) indicated that BCL‐x_L_ levels in MDA‐MB‐231, 4T1, and T47D cells were decreased dramatically after m‐PROTAC‐2 treatment. Furthermore, BCL‐x_L_ levels decreased with increasing m‐PROTAC‐2 concentration (Figures S11A and S12), thus indicating that m‐PROTAC‐2 efficiently induced BCL‐x_L_ degradation in a dose‐dependent manner. Meanwhile, the addition of the proteasome inhibitor MG‐132 to MDA‐MB‐231 cells was found to abolished the m‐PROTAC‐2‐induced decrease in BCL‐x_L_ level, thus confirming that the mechanism underlying the observed decrease in BCL‐x_L_ level involved cellular UPS activity as the same as p‐PROTACs (Figure S11B).

### 2.6 m‐PROTAC‐2 inhibits tumor growth in vivo

BCL‐x_L_ protein is a critical target for overcoming cancer therapy. To confirm the antitumor ability of m‐PROTAC, a xenograft experiment was conducted using BCL‐x_L_ overexpressing 4T1 cells. For in vivo experiments involving mRNA, the delivery system is a significant challenge. In this study, we selected cationic lipid nanoparticles (LNPs) SM‐102 as the delivery vehicle. Initially, luciferase mRNA encapsulated by SM‐102 was injected intratumorally to observe the expression of luciferase in mice through bioluminescence signal. The results demonstrated clear luciferase signals in mice of the mRNA‐LNPs group, indicating the effective transfection of mRNA by LNPs. After 6 h, the signal distribution in the tumor and major organs (heart, liver, spleen, lung, and kidney) was analyzed, revealing that mRNA was only efficiently delivered and expressed within the tumor (Figure S13).

**FIGURE 6:**
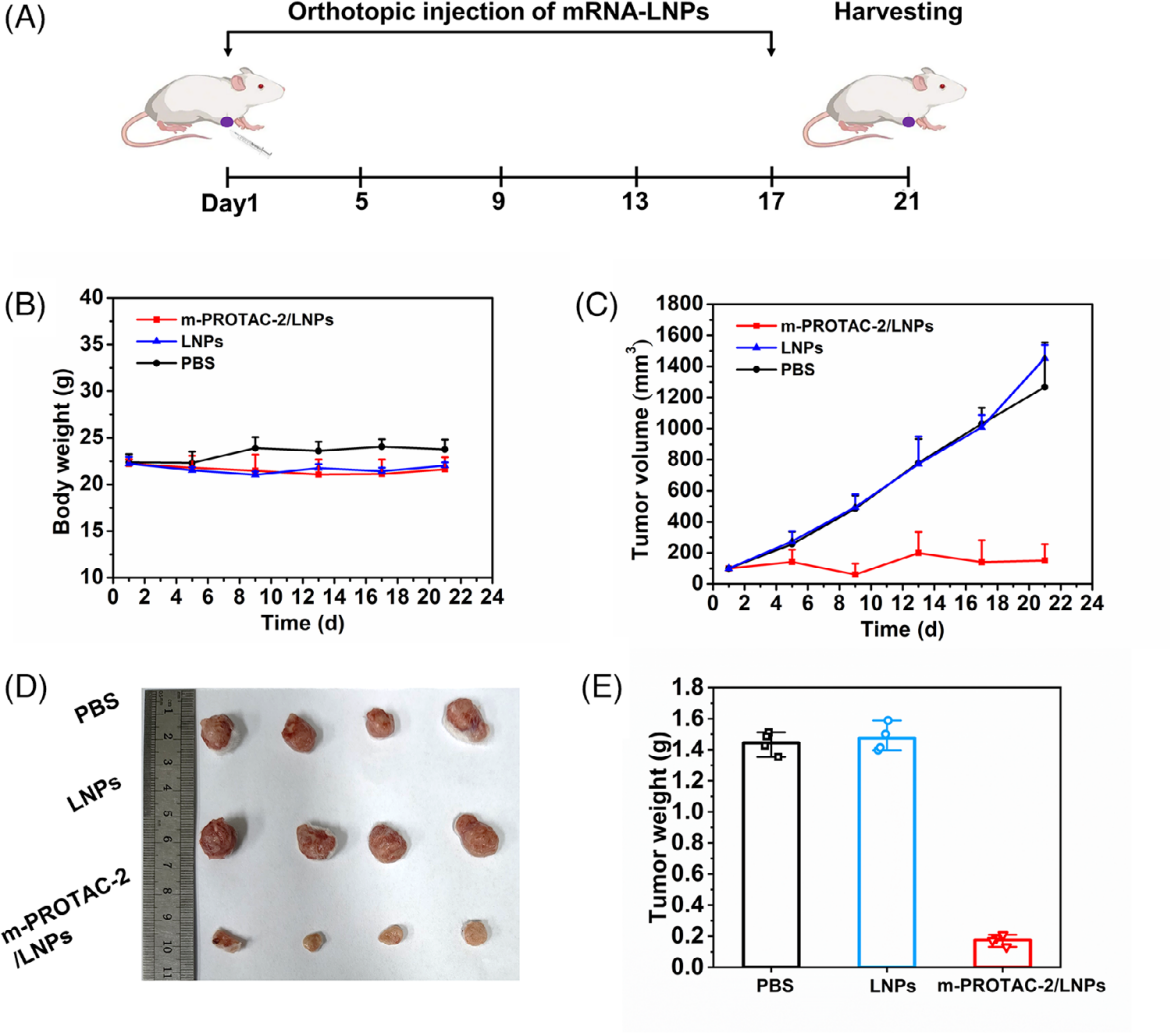
Antitumor activity of mRNA proteolysis‐targeting chimera (m‐PROTAC)‐2 in 4T1 xenograft animal model. (A) The treatment in vivo. (B) Body weight and (C) tumor volume of 4T1 mice model over time when treated with m‐PROTAC‐2/lipid nanoparticles (LNPs) (0.5 mg/kg), LNPs or phosphate‐buffered saline (PBS). (D) Photos and (E) tumor weight of tumors separated from mice. The data are expressed as the mean ± SEM. *n* = 4; ^*^ *p* < 0.05; ^**^ *p* < 0.01; ^***^ *p* < 0.005.

Furthermore, we constructed and characterized LNPs encapsulating m‐PROTAC‐2 (Figure S14), and investigated its antitumor efficacy in vivo. The tumor‐bearing mice were then randomly divided into three groups and injected intratumorally with 0.5 mg/kg m‐PROTAC‐2/LNPs, LNPs vehicle, and phosphate‐buffered saline (PBS) every 4 days, respectively (Figure 6A). In comparison to the control groups treated with LNPs vehicle and PBS, the m‐PROTAC‐2/LNPs group exhibited stable body weight throughout the experiment and no significant variation, while the tumor growth of the m‐PROTAC‐2/LNPs group was dramatically inhibited (Figure 6B,C). This suggests that m‐PROTAC‐2 has low toxicity and a high antitumor ability. Upon dissection, the tumors of the mice treated with m‐PROTAC‐2/LNPs were found to be significantly smaller than those of the control groups (Figure 6D,E). Furthermore, the effective dose of m‐PROTAC‐2 in vivo is significantly lower than that of DT2216 (a novel BCL‐x_L_‐specific PROTAC at 15 mg/kg),^35^, ^36^ while showing superior treatment outcomes. These findings demonstrated the effectiveness of m‐PROTAC in vivo and its significant potential in disease treatment.

## 3 DISCUSSION

In conclusion, here we present for the first time a novel m‐PROTACs strategy which can be constructed through genetic engineering. To verify the strategy, we designed m‐PROTAC‐1 targeting ERα and m‐PROTAC‐2 targeting BCL‐x_L_. Firstly, through fluorescence activated cell sorting and cell imaging experiments, we tested that p‐PROTACs were produced by cytoplasmic translation. And the target POI undergone degradation as a result of p‐PROTACs generation. Subsequently, we determined that m‐PROTACs induced target POIs in a dose‐ and time‐dependent manner. We conducted a series of experiments to confirm that the target protein is degraded through the UPS. Finally, we investigated the in vitro and in vivo antitumor activity of m‐PROTACs, revealing its superior efficacy in inhibiting tumor growth.

m‐PROTACs strategy integrates the advantages of p‐PROTACs and IVT‐mRNA technologies, therefore, exhibits improved biocompatibility, selectivity, and safety, compared to small‐molecule PROTACs and p‐PROTACs. This approach efficiently degrades proteins and has the potential to become an important form of BioPROTACs with more flexible forms.

This strategy offers a genetic engineering approach to construct PROTAC molecules, avoiding the laborious process of chemical synthesis. Indeed, in this strategy, m‐PROTACs rely on the availability of the corresponding peptide ligands. As long as these ligands are accessible, it is theoretically possible to generate the desired m‐PROTACs for targeted proteins degradation. This flexibility and engineering design allows for the potential application of various peptide ligands, expanding the scope and versatility of this approach. Numerous naturally occurring peptide ligands can be applied in this strategy, making it highly adaptable to other E3 ligases and POIs.

As is known to all, traditional gene regulation technologies, such as classical antisense technology, RNA interference (RNAi), and CRISPR/Cas9 technology, primarily regulate protein expression by targeting the gene sequence. The TPD technology based on gene therapy need relatively short time in designing siRNA or guide RNA, and could target large protein libraries. Therefore, they do not have the inherent ability to distinguish between different conformations or post‐translational modifications of the proteins. While, m‐PROTACs strategy is based on the recognition of protein conformations and ligands, which effectively silence proteins with specific conformations or post‐translations. PROTACs, by directly recognizing the target protein, exert their effects more quickly, which displays a distinct advantage in terms of kinetics, enabling the study of acute protein loss. When it comes to long‐lived proteins, m‐PROTAC could get an overall degradation more easily. This strategy may be regarded as a supplement to traditional gene regulation technologies. However, each approach has its advantages and limitations.

Although m‐PROTACs as a new strategy has worked well in vitro and in vivo, it has certain limitations and challenges. For mRNA‐based therapeutics, the most significant challenge lies in delivery system, necessitating the development of effective and novel mRNA delivery methods. The design and optimization of mRNA sequence to enhance transfection efficiency and stability and a deeper understanding of the target degradation mechanisms involved are required. Off‐target effects and specificity are all critical issues that need to be addressed.

However, we believe that as mRNA‐based therapeutics advancing, these challenges will be alleviated or resolved to some extent. The diverse modes of PROTACs will facilitate the development of PROTAC technology, enabling its application to a wider range of “undruggable” targets and diseases.

## 4 MAERIALS AND METHODS

### 4.1 Synthesis of m‐PROTACs

m‐PROTACs were generated by T7 polymerase‐based in vitro transcription. The open‐reading frame of the gene of m‐PROTACs was cloned into the plasmid purchased from VectorBuilder (Guangzhou). The plasmid was linearized with AscI (New England Biolabs) restriction enzyme, and purified using a DNA purification kit (Tiangen). RiboMAXTM Large Scale RNA Production Systems (Promega) were used to generate m‐PROTACs, adding the m7GpppG cap structure using Ribo m7G Cap Analog (Promega). The prepared m‐PROTACs were further purified by RNA purification kit (Tiangen). The final products were quantified by Nanodrop 2000 (ThermoFisher Scientific) and stored at −193 K.

### 4.2 Cell culture

MCF‐7, T47D, 4T1, and MDA‐MB‐231 cells were purchased from the Shanghai Cell Bank of the Chinese Academy of Sciences. All the medium was supplemented with 10% fetal bovine serum (FBS), penicillin (100 U/mL), and streptomycin (100 μg/mL). MCF‐7 and T47D cells were grown in Dulbecco's modified Eagle's medium (DMEM), 4T1 cells were grown in DMEM/F12 medium, and MDA‐MB‐231 cells were grown in Leibovitz's L‐15 medium. MCF‐7, T47D, and 4T1 cells were incubated at 310 K in a humidified atmosphere containing 5% CO_2_ as a reserve resource. MDA‐MB‐231 cells were incubated at 310 K in a humidified atmosphere without CO_2_. Subcultivation was done every 2−3 days.

### 4.3 Transfection of m‐PROTACs

To perform transfection of cells, cells were seeded into confocal dishes or 12‐well plates, and cultivated overnight at 310 K. Next day, according to manufacturer's instructions, per μg of m‐PROTACs were mixed with 2 μL lipo6000 reagent (Beyotime Biotechnology) and incubated for 15 min at room temperature (RT) to form lipoplexes. The cells were washed with PBS and incubated with the transfection complexes for 4 h in the culture without FBS. After that, the medium was replaced by complete medium and incubated for 2, 8, 20, 32, or 44 h, respectively.

### 4.4 Fluorescence activated cell sorting

Cells were transfected with 0.5 and 1 μg/mL m‐PROTAC‐1 for 24 h. The cells were collected and centrifuged at 1000 *g* for 5 min to remove the supernatant. The cells were resuspended in pre‐cooled PBS. Next, the eGFP fluorescence in the cells was analyzed by flow cytometry (Beckman & Coulter) and FlowJo 10.6. The experiments were repeated three times, and at least 10,000 gated events were collected and analyzed each time.

### 4.5 Western blot assay

Cells were seeded in each well of a 12‐well plate and grown in complete medium overnight. Then, the cells were transfected with serial concentrations of m‐PROTACs for a specific time with or without MG‐132/MLN4924. To extract protein, the treated cells were lysed in RIPA buffer (Beyotime Biotechnology) according to the manufacturer's instructions. Protein concentration was assessed with the BCA protein assay kit (Beyotime Biotechnology). The supernatants (20 μg protein) were separated by 10% Sodium dodecyl sulfate‐polyacrylamide gel electrophoresis (SDS/PAGE) analysis and transferred to the PVDF membrane. Membranes were blocked with 5% milk and washed three times with TBST (containing 0.1% Tween‐20) for 10 min each time, followed by immunoblotting with the anti‐ERα (#8644s, Cell Signaling Technology) or BCL‐x_L_ (31011ES50, Yeasen Biotechnology) and anti‐β‐actin antibodies (#AC026, ABclonal) in 5% Bovine serum albumin (BSA) overnight at 277 K. The PVDF membranes were washed with TBST and incubated with the secondary antibody (A‐0208, Beyotime Biotechnology) in 5% milk for 1 h, and then washed three times with TBST. MG132 (HY‐13259, 10 μM) and MLN4924 (HY‐70062, 0.5 μM) were purchased from MedChemExpress. The gel imager (Tanon 5200) was used to display the image using the clarity western ECL substrate (Biorad). The intensity of the signal was quantified using the ImageJ‐Fiji software. Graphical output and statistical analysis (analysis of variance [ANOVA]) were generated with GraphPad Prism 8 software.

### 4.6 Co‐immunoprecipitation

Cells were seeded in each well of a six‐well plate and grown in complete medium overnight. Then, the cells were transfected with 1 μg/mL m‐PROTAC‐1 for 24 h with 10 μM MG‐132. Whole cellular extracts were prepared in IP lysis buffer including protease inhibitor. The protein G Dynabeads conjugated with anti‐ERα rabbit antibody or normal IgG were added into whole cellular extracts and incubated overnight at 4°C. After washing three times with cold Phosphate Buffered Saline with Tween‐20 (PBST), the binding components were eluted by boiling with SDS–PAGE loading buffer, and were analyzed the levels of ubiquitinated ERα with western blot.

### 4.7 Confocal microscopy imaging

Cells were seeded in a confocal dish (20 mm dish, Biosharp) and incubated overnight. Then, the cells were transfected with m‐PROTAC‐1 for a specific time. For immunofluorescence, the treated cells were fixed in 4% Paraformaldehyde for 15 min, following with 0.25% Triton X‐100 for 5 min. Then, dishes were treated with 1% BSA in PBST at 310 K for 60 min and washed three times with PBST for 5 min. Next, the anti‐ERα antibody at a dilution rate of 1:300 in 1% BSA was incubated with the cells for 1.5 h. After being washed with PBST, cells were incubated with Cy5‐conjugated IgG antibody (#550083, Zen Bioscience) at a dilution rate of 1:1000 in 1% BSA at 310 K for 40 min. Finally, the cells were stained with 4′,6‐diamidino‐2‐phenylindole (DAPI) for 5 min and washed with PBST three times. The image was taken with a confocal laser scanning microscope (NIKON A1 HD25). The intensity of the signal was quantified using ImageJ‐Fiji software. Graphical output and statistical analysis (ANOVA) were generated with GraphPad Prism 8 software.

### 4.8 RNA isolation and qRT‐PCR analysis

A total of 3 × 10^5^ cells were seeded in each well of a six‐well plate and grown in complete medium overnight. Then, the cells were transfected with 1 μg/mL of m‐PROTACs for a specific time. Total RNA was extracted from culture cells using Trizol reagent (15596026, Thermo Fisher) following the manufacturer's instructions. An amount of 2000 ng of total RNA was reverse transcribed using the TransScript All‐in‐One First‐Strand cDNA Synthesis SuperMix (AE341‐03, Trans) for qRT‐PCR. qRT‐PCR was performed using PerfectStart Green qPCR SuperMix (AQ601, Trans) in qTOWER 2.0 (Analytik Jena AG). The thermocycler was set up to run at 95°C for 1 min, 40 cycles of 95°C for 15 s, and 60°C for 45 s (plate read), followed by a 60°C−95°C melting curve. The 2^–ΔΔCt^ method was used to analyze and compare the mRNA expression.

### 4.9 Anti‐proliferation assay

A total of 1 × 10^4^ cells were seeded in each well of the 96‐well plates and allowed to grow overnight. Cells were transfected with 100 μL serial concentrations of m‐PROTAC‐1 complexes at 310 K for 24 or 48 h. At the end of the exposure, the medium was replaced with 100 μL medium adding 10 μL CCK‐8 reagent. After incubating for 1 h, the absorbance (OD) was measured at 450 nm by employing a Microplate Reader (Tecan Infinite). The cell viability was analyzed using GraphPad Prism 8 software.

### 4.10 Cell apoptosis and cycle assay

Cells transfected with m‐PROTAC‐1 at different concentrations for 24 h were collected, then centrifuged at 1000 *g* for 5 min to remove the supernatant. For cell apoptosis analysis, the cells were stained with Annexin V‐FITC and PI following the instructions of Annexin V‐FITC Apoptosis Detection Kit (Beyotime Biotechnology). For cell‐cycle analysis, the cells were pretreated in 70% ice‐cold ethanol at 277 K overnight, then centrifuged. The pretreated cells were stained with PI at the presence of RNase A following the instructions of Cell Cycle and Apoptosis Analysis Kit (Beyotime Biotechnology). Finally, the stained cells were tested using a flow cytometer (CytoFlex, Beckman Coulter) and analyzed using ModFit LT 5.0. For each individual experiment, over 10,000 cells were counted for the cell apoptosis and cycle assay.

### 4.11 Animal experiments

BALB/c mice used in this study were purchased from Guangdong Medical Laboratory Animal Center. Tumor models were established using 4‐week‐old female BALB/c mice by injecting 1 × 10^5^ 4T1 cells per mouse subcutaneously. All animal experiments were conducted according to the guidelines and approval of the Institutional Animal Care and Use Committee of Shenzhen International Graduate School, Tsinghua University, and the Medical Laboratory Animal Center of Guangdong, China (License No. 9 2023).

The tumor‐bearing mice were injected intratumorally with either PBS, LNPs vehicle, or m‐PROTAC‐2/LNPs. For preparation of m‐PROTAC‐2/LNPs, the lipid mixture was prepared by dissolving cationic liposomes SM‐102, distearoylphosphatidylcholine, cholesterol, and DMG‐PEG2000 in ethanol at a molar ratio of 50:10:38.5:1.5. Additionally, firefly luciferase mRNA or m‐PROTAC‐2 was diluted in 50 mM citrate buffer to obtain an aqueous solution of mRNA. Briefly, mRNA/LNPs were formed by mixing the lipid mixture with the mRNA aqueous solution at a volume ratio of 1:3 using a microfluidic device (nanoE, Micro&Nano) and then dialyzed against PBS (pH 7.4) for 18 h to remove ethanol and complete the citrate buffer exchange process. mRNA/LNPs size distribution, polydispersion index, and electrical potential (Zeta) were determined by Litesizer 500 instrument (Anton Paar, Austrian). The encapsulation efficiency of LNPs was assessed using the RiboGreen method.

For transfection of luciferase mRNA‐LNPs, the mice were injected with luciferase mRNA or PBS after the tumor‐bearing mice model was successfully established, and the signals were recorded by small animal live imaging instrument (IVIS Spectrum, PerkinElmer) at 3 or 6 h after injection. One of the mice from each group was dissected after 6 h, and tumors and organs were harvested for ex vivo imaging (In‐Vivo F Pro, Bruker).

To validate the efficacy of m‐PROTAC in vivo, mice with tumors of approximately 100 mm^3^ were randomly divided into three groups. The tumor‐bearing mice were injected intratumorally with either PBS, LNPs vehicle, or m‐PROTAC‐2/LNPs (0.5 mg/kg) every 4 day. Tumor volume and mice weight were measured before each injection and calculated using the formula *V* = 1/2*ab* ^2^ (where *a* is the largest diameter and *b* is the smallest diameter). Mice were continued to be monitored for 4 days after the last injection and then treated with CO_2_ euthanasia. The tumor and multiple organs were removed, and the tumor was weighed and the volume measured.

### 4.12 Statistical analysis

The data are presented as mean values ± standard error of mean (SEM). Statistical analysis for comparing two experimental groups was performed using one‐sided Student's *t*‐tests. A value of *p* < 0.05 was considered to be significant. Analyses were performed with Prism 8 (GraphPad Software). Differences are labeled n.s. for not significant, * for *p* ≤ 0.05, and ** for *p* ≤ 0.01. Preestablished criteria for the removal of animals from the experiment were based on animal health, behavior, and well‐being as required by ethical guidelines.

## AUTHOR CONTRIBUTIONS

X. X., C. Z., and X. L. contributed equally to this work. X. X. and Y. T. proposed the idea. X. X., C. T., Y. J., and Y. T. designed the experiments. C. Z., J. W., and H. L. contributed to the animal experiments. X. L. and H. Z. helped with the experiments of qPCR. N. X. and Y. F. also provided help in the cell experiments. X. X. and Y. T. wrote the manuscript. All the authors have read and approved the final manuscript.

## CONFLICT OF INTEREST STATEMENT

Hongyan Li is an employee in Shenzhen NeoCura Biotechnology Co., other authors have no potential relevant financial or non‐financial interests to disclose.

## ETHICS STATEMENT

BALB/c mice were purchased from the Guangdong Medical Laboratory Animal Center. All animal experiments were conducted according to the guidelines and approval of the Institutional Animal Care and Use Committee of Shenzhen International Graduate School, Tsinghua University, and the Medical Laboratory Animal Center of Guangdong, China (License No. 9 2023).

## Supporting information

Supporting Information

## Data Availability

The data that support the finding of this study are available from the corresponding author upon reasonable request.
