# Supplementary material for: mRNA PROTACs: engineering PROTACs for high‐efficiency targeted protein degradation
Source: MedComm (2020). 2024 Feb 19;5(2):e478. doi: 10.1002/mco2.478 (PMC10876204; doi:10.1002/mco2.478)
Supplement: Supplementary file 1 — Supporting Information [file MCO2-5-e478-s001.docx]

**Supporting Information**

**For**

**mRNA PROTACs: Engineering PROTACs for high-efficiency targeted protein degradation**

Xiaoqi Xue^1,#^, Chen Zhang^1,#^, Xiaolin Li^1,#^, Junqiao Wang^1^, Haowei Zhang^1^, Ying Feng^1^, Naihan Xu^1,2^, Hongyan Li^3^, Chunyan Tan^1^, Yuyang Jiang^1^, Ying Tan^1,^[[1]](#footnote-1)^*^

^1^ State Key Laboratory of Chemical Oncogenomics, Institute of Biopharmaceutical and Health Engineering, Shenzhen International Graduate School, Tsinghua University, Shenzhen, 518055, China

^2^ School of Food and Drug, Shenzhen Polytechnic University, Shenzhen, 518055, China

^3^ Shenzhen NeoCura Biotechnology Co., Ltd, Shenzhen 518055, China


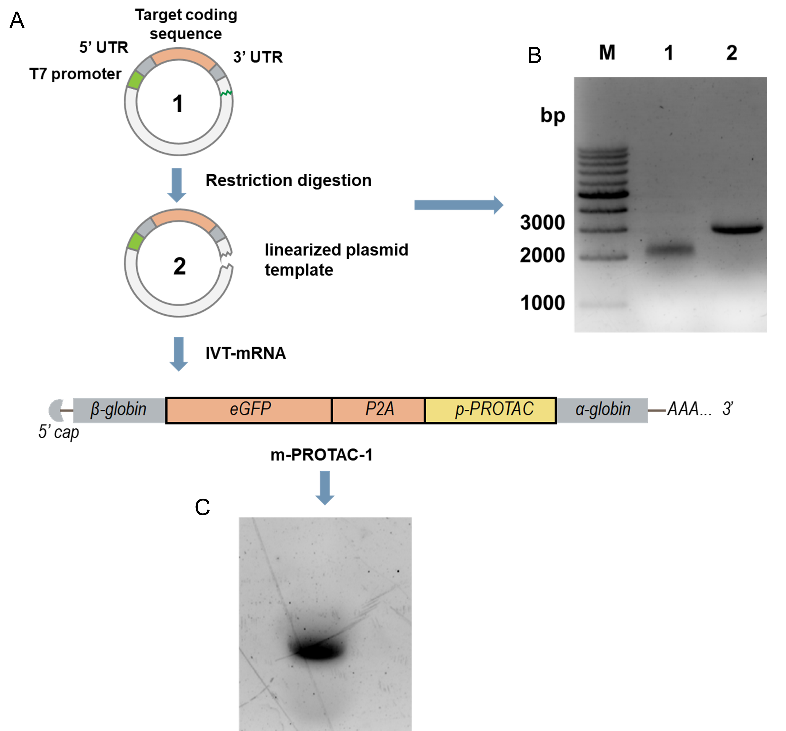


**FIGURE S1.** (A) Synthesis of m-PROTAC-1. (B) The result of the 1% agarose gel of plasmid and the linearized plasmid template. (C) The result of the 1% agarose gel of m-PROTAC-1.

*
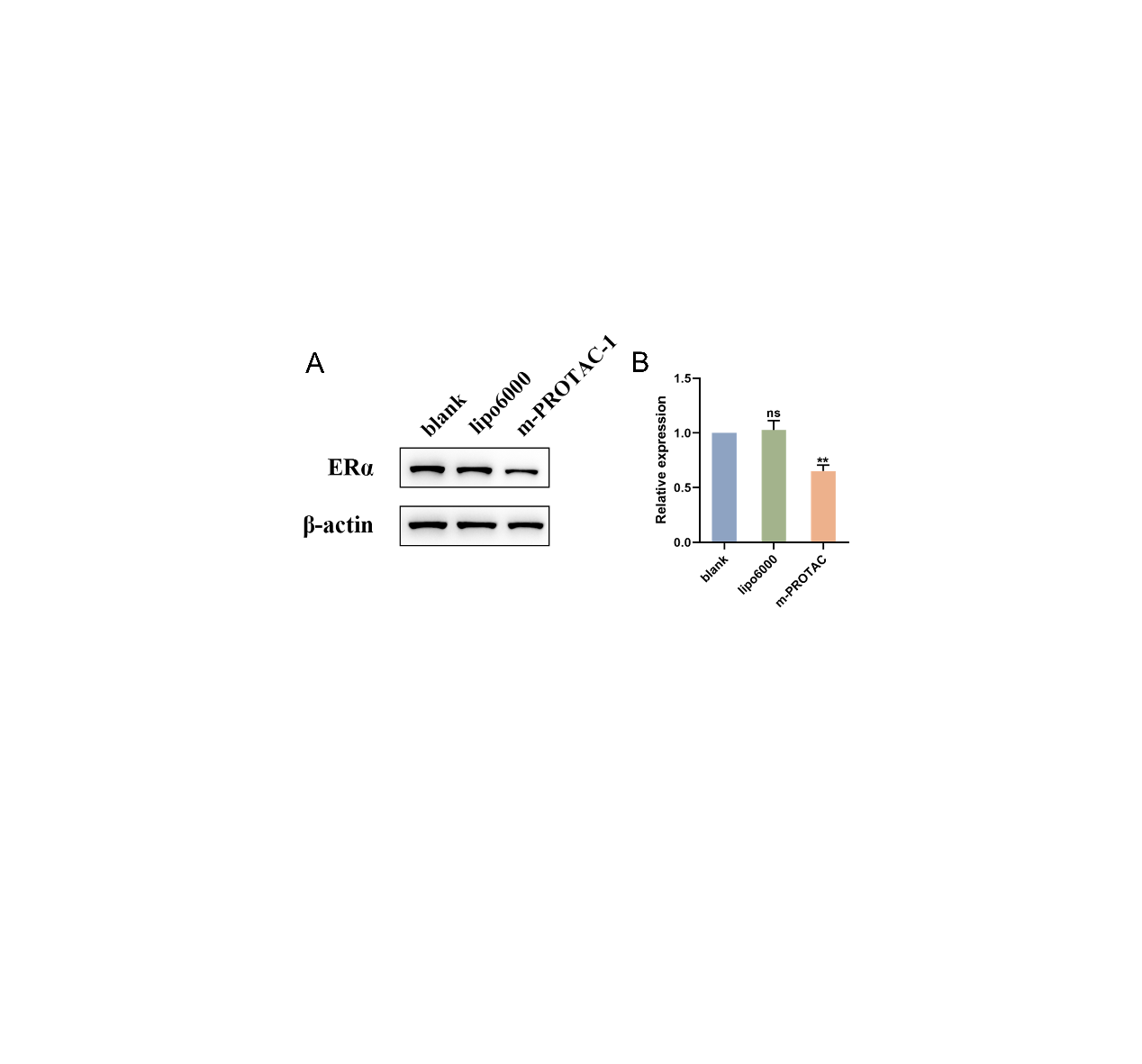
*

**FIGURE S2.** (A) Western blot of ERα in MCF-7 cells after transfected with only lipo6000 or 1 μg/mL m-PROTAC-1 lipo6000 complex for 24 h. (B) Quantification of expression levels of ERα of MCF-7 cells from A.


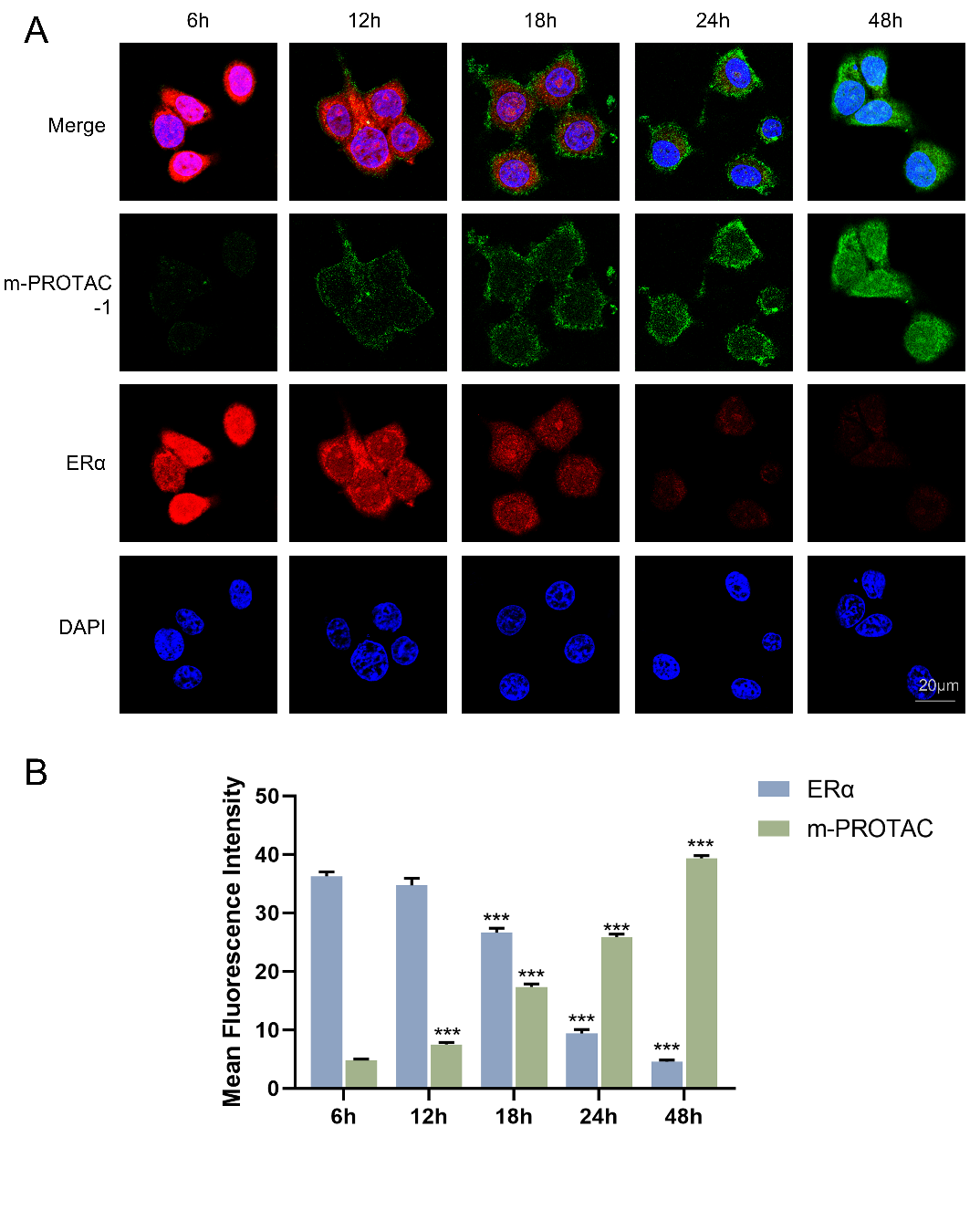


**FIGURE S3.** (A) Confocal microscopy images of MCF-7 cells treated with 1 μg/mL for 6h, 12h,18h, 24h and 48h (scale bar, 20 μm). (B) Quantification of relative ERα and m-PROTAC-1 expression level of MCF-7 cells from A. Data are presented as means ± SEM of triplicate independent experiments for (B) (*n*=3). **P* < 0.05. ***P* < 0.01.


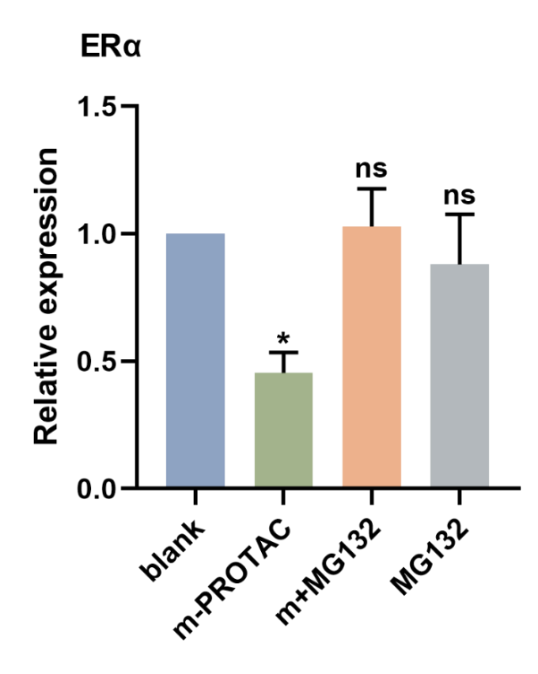


**FIGURE S4.** Quantification of expression levels of ERα in MCF-7 cells from FIGURE 4A. Data are presented as means ± SEM of triplicate independent experiments (*n*=3).


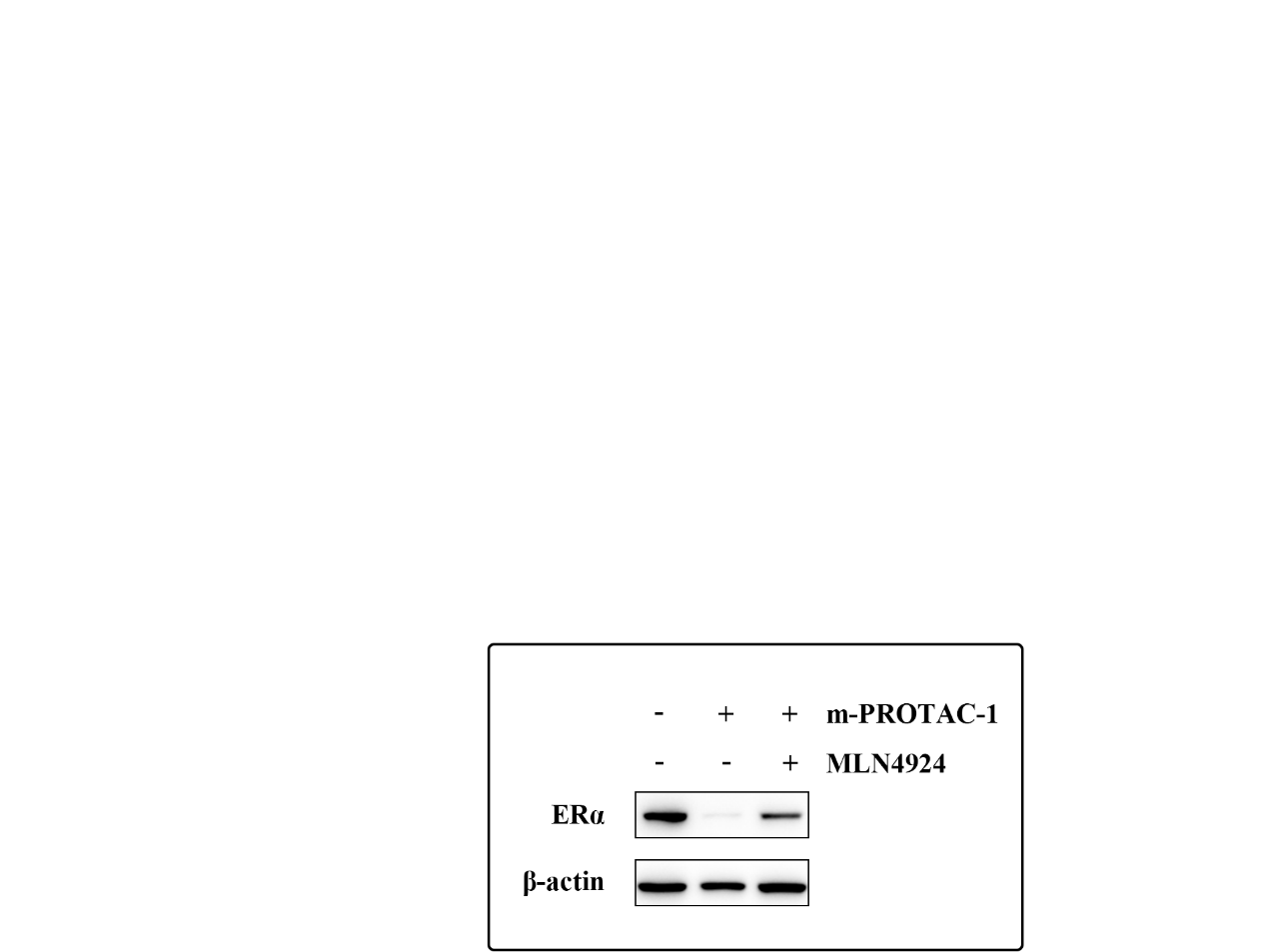


**FIGURE S5.** Western blot of ERα in MCF-7 cells after transfected with 1 μg/mL m-PROTAC-1 with or without 0.5 μM MLN4924 for 24 h.

*
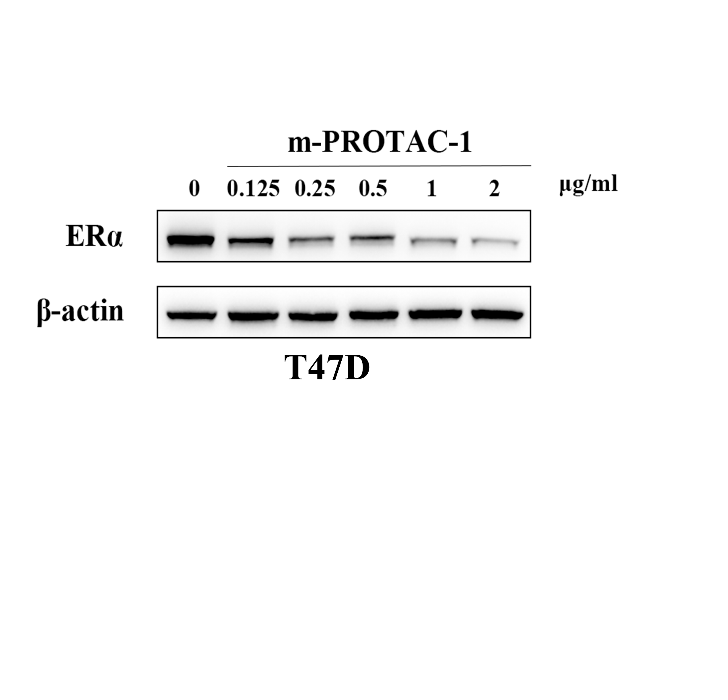
*

**FIGURE S6.** Western blot of ERα in T47D cells after transfected with the indicated concentrations of m-PROTAC-1 for 24 h.

**Table S1.** Sequences of used primers for qRT-PCR

| Primer | Forward （5’-3’） | Reverse （5’-3’） |
| --- | --- | --- |
| ESR1 | CCCACTCAACAGCGTGTCTC | CGTCGATTATCTGAATTTGGCCT |
| GAPDH | ATCAAGAAGGTGGTGAAGCA | AAGGTGGAGGAGTGGGTGT |

*
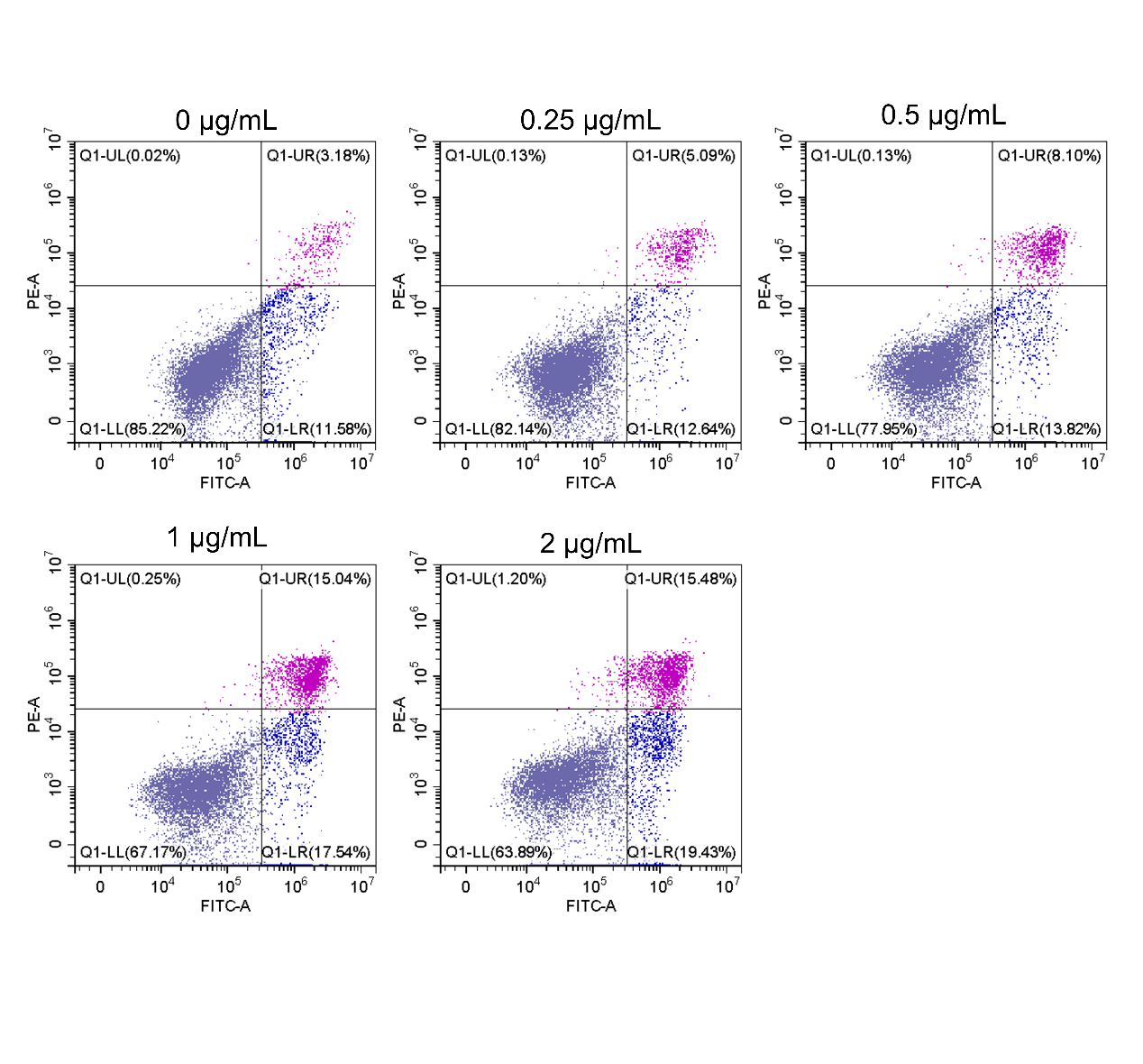
*

**FIGURE S7.** Flow cytometry analysis of Annexin V-FITC Apoptosis Detection Kit of apoptotic

cells following m-PROTAC-1 treatment to MCF-7 cells with indicated concentrations for 24 h.


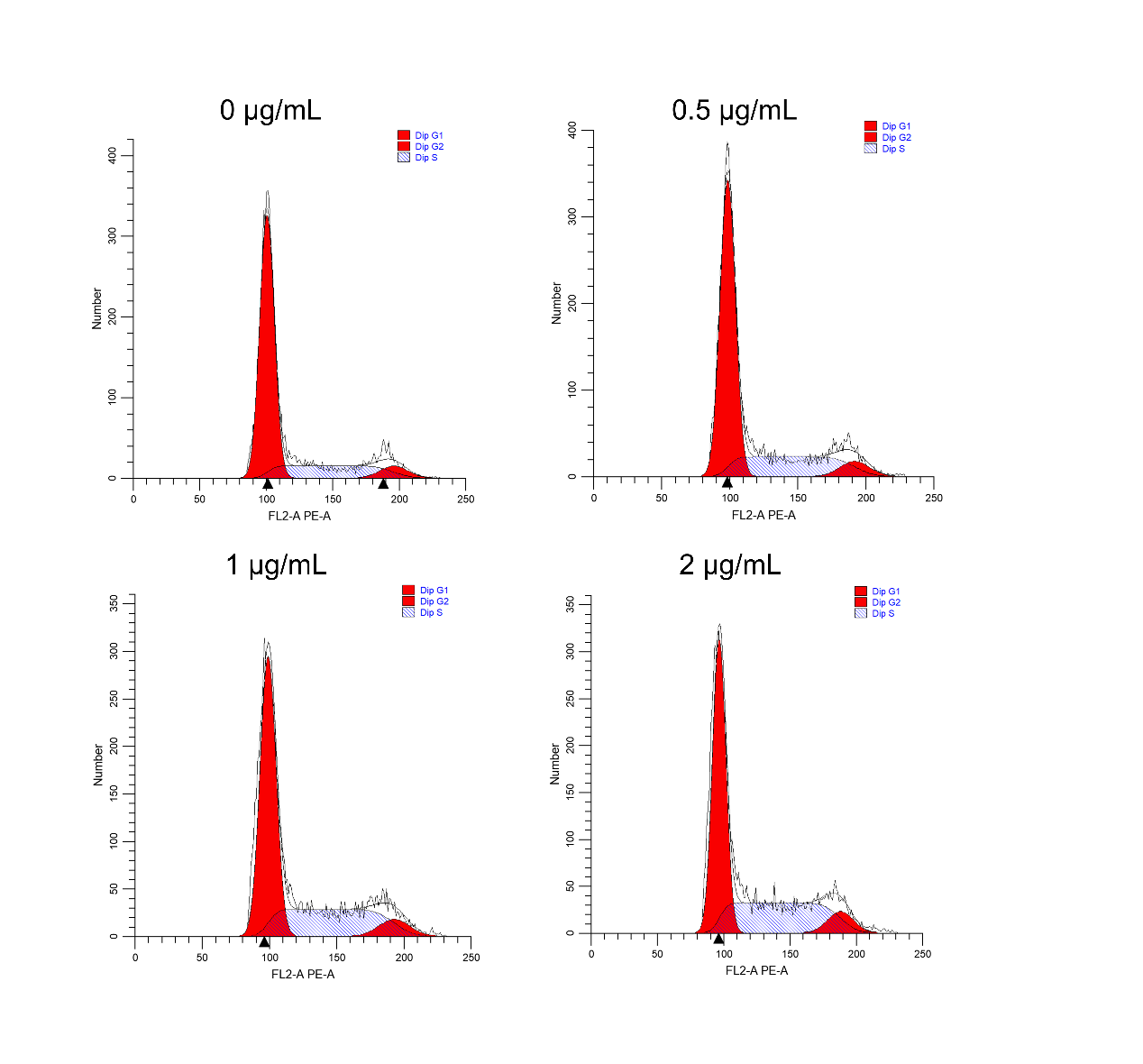


**FIGURE S8.** Flow cytometry analysis of the cell cycle distribution following m-PROTAC-1 treatment to MCF-7 cells with indicated concentrations for 24 h.


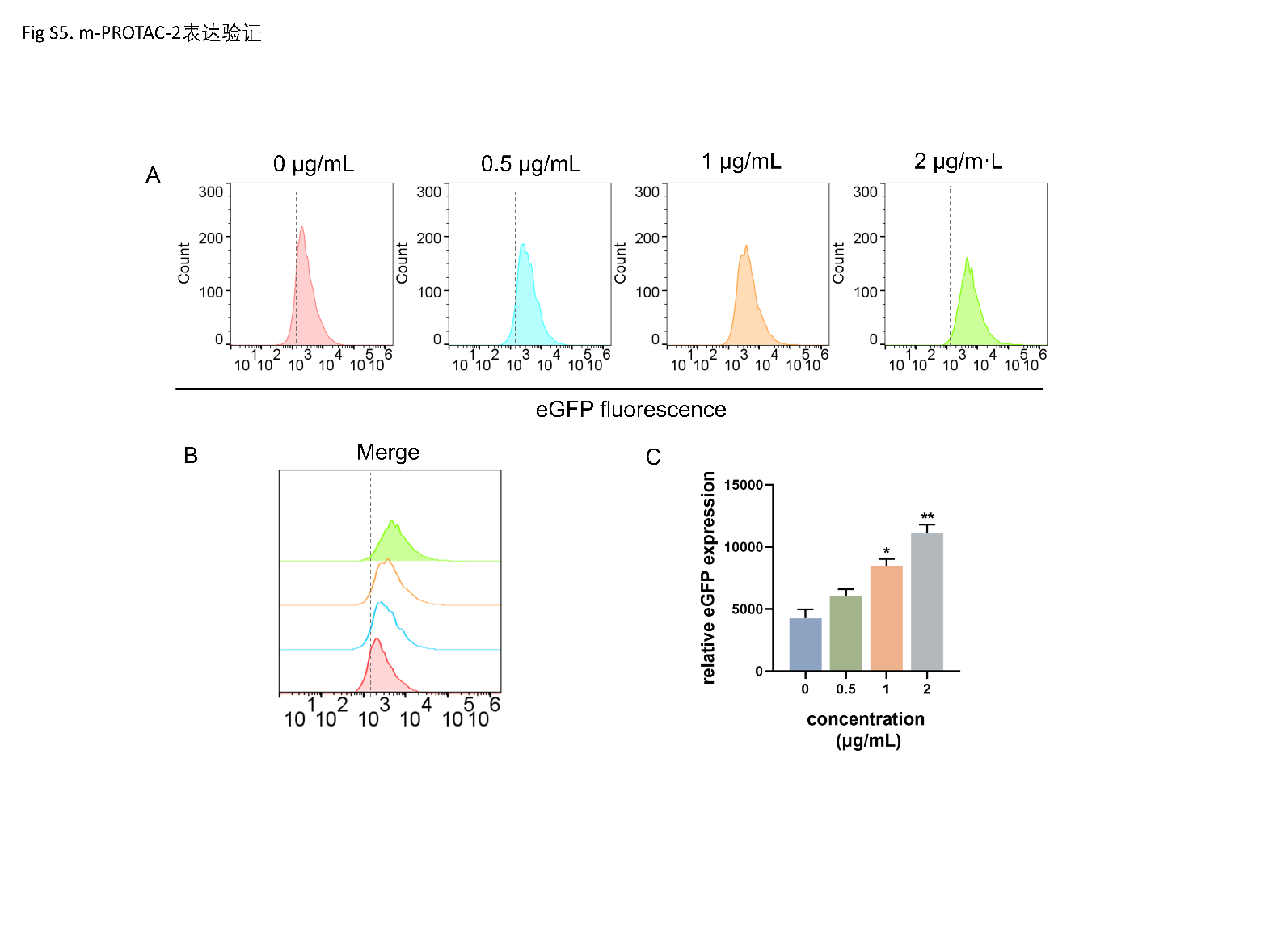


**FIGURE S9.** m-PROTAC-2 showed stable translation efficiency in MDA-MB-231 cells. (A) Flow cytometry analysis (histogram) of MDA-MB-231 cells transfected with the indicated concentrations of m-PROTAC-2 for 24 h and (B) comparison of the histogram at different concentration from A. (C) Quantification of relative eGFP expression level of MDA-MB-231 cells from A. Data are presented as means ± SEM of triplicate independent experiments (*n*=3).

*
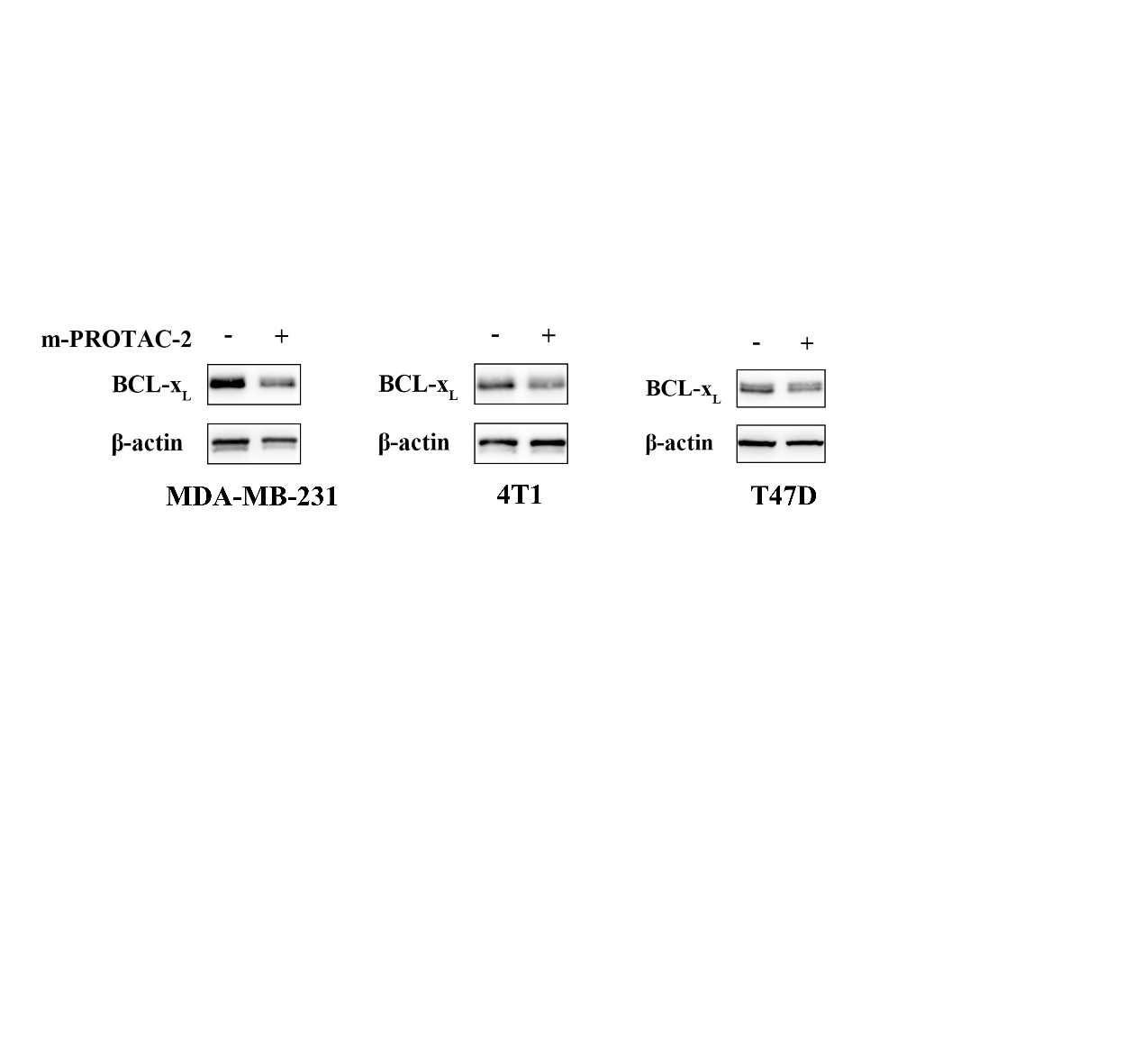
*

**FIGURE S10.** Western blot of BCL-x_L_ in different cell lines after transfected with 1 μg/mL m-PROTAC-2 for 24 h.

**
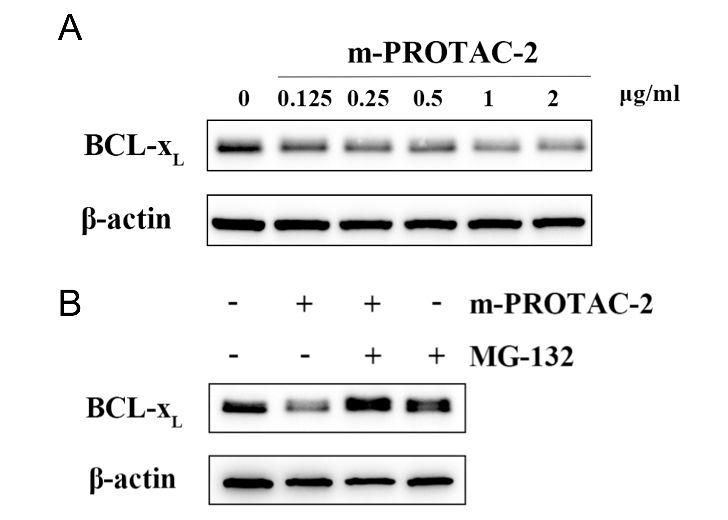
**

**FIGURE S11.** m-PROTAC-2 induced the degradation of BCL-x_L_ in MDA-MB-231 cells. (A) Western blot of BCL-x_L_ transfected with indicated concentrations of m-PROTAC-2 for 24 h. (B) Western blot of BCL-x_L_ transfected with 1 μg/mL m-PROTAC-2 with or without 10 μM MG-132 for 24h.


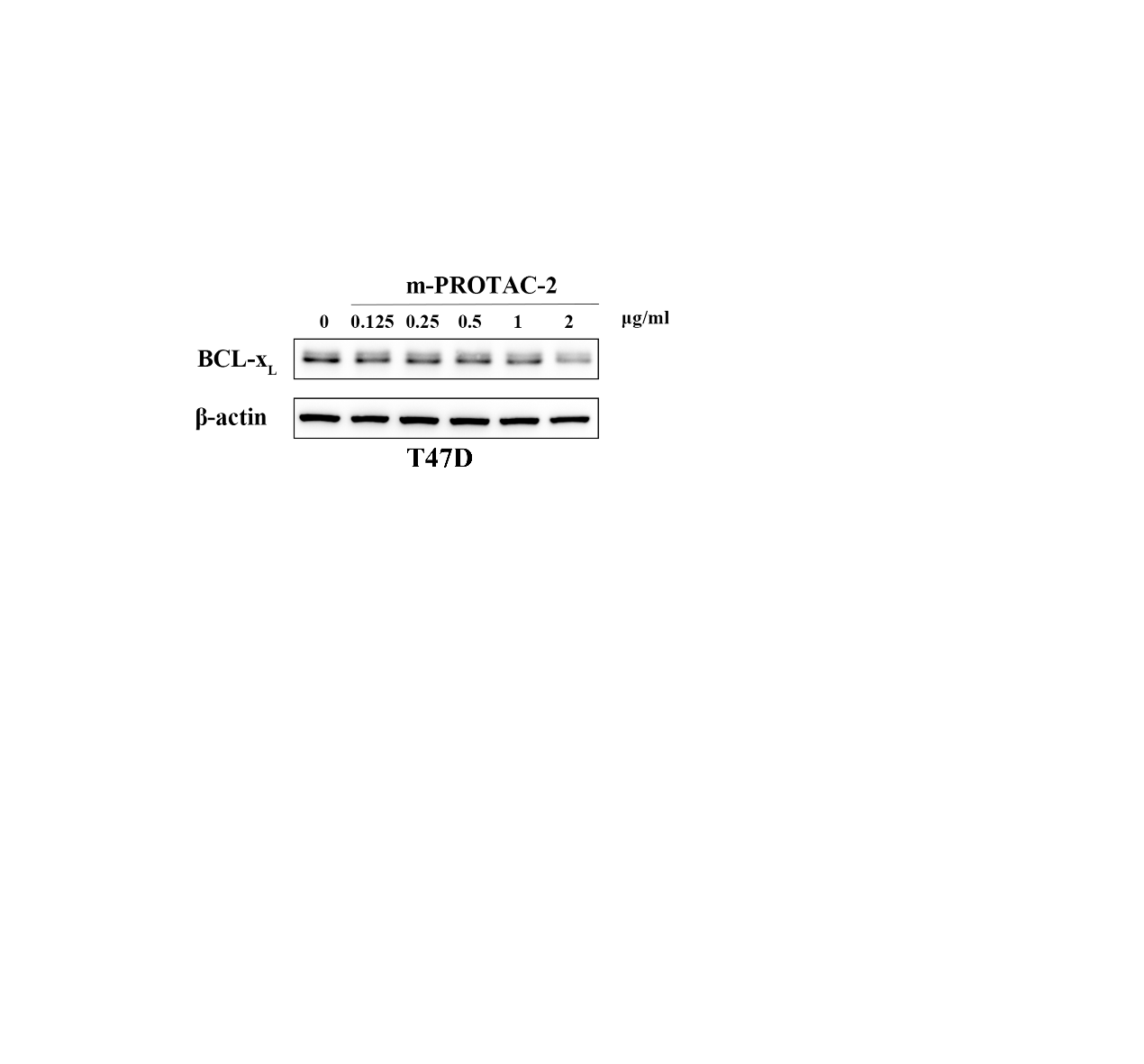


**FIGURE S12.** Western blot of BCL-x_L_ in T47D cells after transfected with the indicated concentrations of m-PROTAC for 24 h.


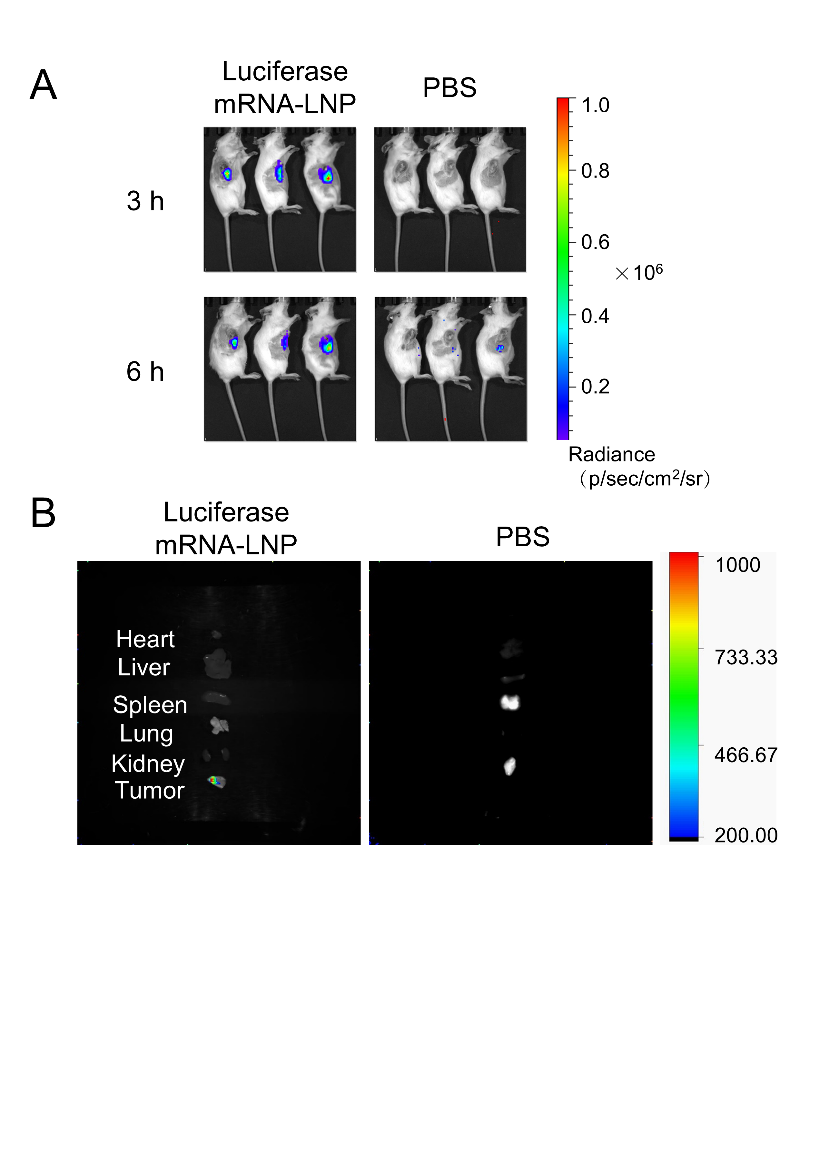


**FIGURE S13.** (A) In vivo bioluminescence images of mice after treated with Luciferase mRNA-LNPs or PBS 3 h or 6 h. (n=3.) (B) Ex *vivo* bioluminescence images of tumors and major organs after treated with Luciferase mRNA-LNPs or PBS for 6 h.


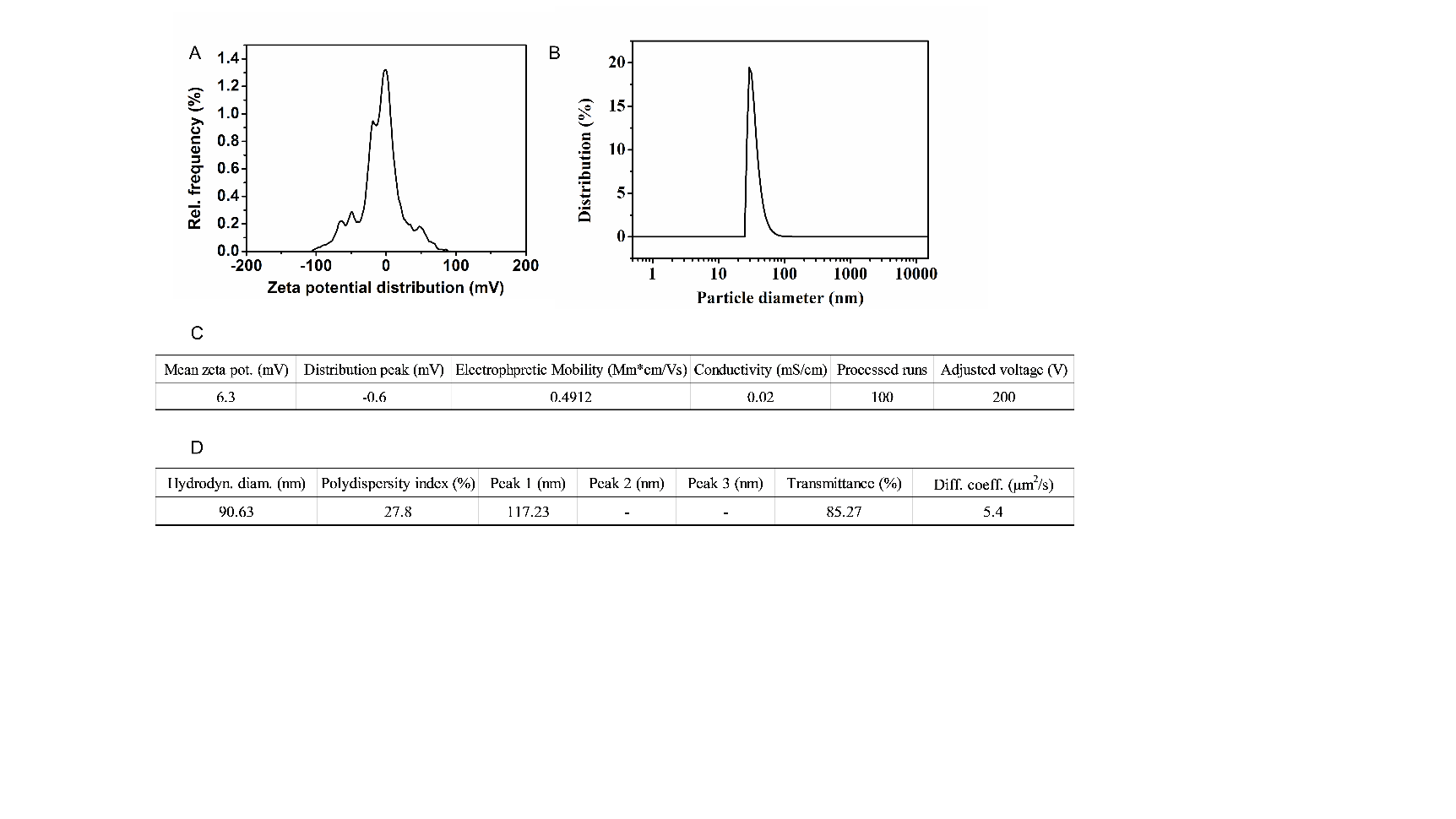


**FIGURE S14.** (A)Zeta potential distribution of m-PROTAC-2/LNPs (B) Particle size distribution (intensity) of m-PROTAC-2/LNPs (C) Measurements of zeta potential analysis (D) Measurements of particle size analysis.

1. [↑](#footnote-ref-1)
